# Barriers and Facilitators to Safe Food Handling among Consumers: A Systematic Review and Thematic Synthesis of Qualitative Research Studies

**DOI:** 10.1371/journal.pone.0167695

**Published:** 2016-12-01

**Authors:** Ian Young, Lisa Waddell

**Affiliations:** 1 School of Occupational and Public Health, Ryerson University, Toronto, Ontario, Canada; 2 National Microbiology Laboratory @ Guelph, Public Health Agency of Canada, Guelph, Ontario, Canada; Agricultural University of Athens, GREECE

## Abstract

Foodborne illness has a substantial health and economic burden on society, and most cases are believed to be due to unsafe food handling practices at home. Several qualitative research studies have been conducted to investigate consumers’ perspectives, opinions, and experiences with safe food handling at home, and these studies provide insights into the underlying barriers and facilitators affecting their safe food handling behaviours. We conducted a systematic review of previously published qualitative studies in this area to synthesize the main across-study themes and to develop recommendations for future consumer interventions and research. The review was conducted using the following steps: comprehensive search strategy; relevance screening of abstracts; relevance confirmation of articles; study quality assessment; thematic synthesis of the results; and quality-of-evidence assessment. A total of 39 relevant articles reporting on 37 unique qualitative studies were identified. Twenty-one barriers and 10 facilitators to safe food handling were identified, grouped across six descriptive themes: confidence and perceived risk; knowledge-behaviour gap; habits and heuristics; practical and lifestyle constraints; food preferences; and societal and social influences. Our overall confidence that each barrier and facilitator represents the phenomenon of interest was rated as high (n = 11), moderate (11), and low (9). Overarching analytical themes included: 1) safe food handling behaviours occur as part of a complex interaction of everyday consumer practices and habituation; 2) most consumers are not concerned about food safety and are generally not motivated to change their behaviours based on new knowledge about food safety risks; and 3) consumers are amenable to changing their safe food handling habits through relevant social pressures. Key implications and recommendations for research, policy and practice are discussed.

## Introduction

Foodborne disease has a substantial global burden on morbidity and mortality [1]. In the United States (US), foodborne disease agents are estimated to cause 48 million cases of illness each year, resulting in approximately 128,000 hospitalizations and 3,000 deaths [2,3]. In Canada, approximately 4 million cases of domestically-acquired foodborne illness occur each year, resulting in an estimated 11,600 hospitalizations and 238 deaths [4,5]. These illnesses have significant economic impacts on society through direct healthcare costs and indirect costs such as lost productivity [6], and they occasionally also result in costly food recalls and trade disruptions.

The specific proportion of foodborne disease that can be attributed to household preparation and consumption of food is unknown due to a lack of consistent and routine reporting of illnesses from such settings and limited mechanisms to capture these data within current surveillance systems. However, previous research suggests that most sporadic cases of enteric illness are associated with exposure at home vs. other settings [7–9], and most foodborne disease outbreaks in Europe are associated with domestic household settings [10]. Several previous qualitative research studies have been conducted to investigate consumers’ perspectives, opinions, and experiences with safe food handling at home [11–13]. These studies provide insights into the underlying reasons affecting consumers’ adoption and maintenance of their behaviours, which can help to guide the development of appropriate behaviour change interventions and other policy actions [14,15].

The purpose of this study was to conduct a systematic review and thematic synthesis of qualitative primary research studies investigating the barriers and facilitators to safe food handling among consumers, with the goal of developing overarching themes and new interpretations of the results [15,16]. To date, no previous studies have used structured and transparent knowledge synthesis methods to identify, evaluate, and synthesize the across-study themes from the qualitative research literature in this area. The results of this synthesis can be used by food safety decision-makers and practitioners who are responsible to inform the design and delivery of future safe food handling interventions for consumers.

## Materials and Methods

### Review approach, question, and eligibility criteria

This review was conducted following recommended steps for systematic reviews of qualitative research [16,17], and was reported following the “Enhancing Transparency in Reporting the Synthesis of Qualitative Research (ENTREQ)” guidelines [18]. The review protocol (which contains copies of all forms used) and a copy of the ENTREQ checklist are available as supplementary information (S1 File and S1 Table). The review question was: “What are the barriers and facilitators to safe food handling among consumers in developed countries?” The population of interest was adult consumers (≥18 years old) who prepare or handle food for consumption at home. The outcome of interest was safe food handling (including personal hygiene, avoiding cross-contamination, adequate cooking, keeping food at safe temperatures, and avoiding “risky” food consumption) in relation to microbial food safety [19]. Only research conducted in countries classified as “very high human development” by the United Nations Development Programme was considered in this review, as studies in these settings were most relevant to our end-users (Canadian food safety decision-makers and practitioners). Any qualitative or mixed-method primary research study published in English, French, or Spanish was considered for inclusion.

### Search strategy

A scoping review was conducted by the authors in 2014 that identified a list of 86 qualitative research studies related to food safety education of consumers [11]; this list was used as a starting point for the search strategy. An updated and modified search was conducted during January 6–8, 2016, in the same 10 bibliographic databases used in the previous scoping review: Scopus, PubMed, Agricola, CAB Abstracts, Food Safety and Technology Abstracts, PsycINFO, Educational Resources Information Center, Cumulative Index to Nursing and Allied Health Literature, ProQuest Public Health, and ProQuest Dissertations and Theses [11]. The original search algorithm included a combination of food safety-related terms (e.g. food safety), population terms (e.g. consumers, adults), intervention terms (e.g. program, course, campaign), and outcome terms (e.g. behavior, knowledge, attitudes). In the updated search, the category of “intervention” terms was removed, some population key words were modified, and another category of “qualitative research” terms (e.g. qualitative, focus groups) was added. The modified algorithm was pre-tested in Scopus prior to implementation to ensure that it would capture all of the known 86 qualitative research articles.

Complementary searches were conducted in Google to identify grey literature (e.g. research reports) using simple search strings (e.g. “consumer food safety focus groups”). Additionally, the reference lists of all relevant articles and six relevant literature reviews were reviewed to identify additional potentially relevant articles. Additional details on the search strategy, including full copies of all search algorithms used, is available as supplementary information (S2 File).

### Relevance screening, data extraction, and quality assessment

The titles and abstracts of all identified references were screened using a pre-specified form containing one question to determine whether the study met the eligibility criteria. Full papers of relevant articles were then obtained and confirmed for relevance using another pre-specified form. The form was used to extract key characteristics from relevant articles, including: study aim and location, target population socio-demographics, study methodology, and data collection procedures. All relevant studies were then critically appraised using a quality assessment form containing eight criteria. The criteria were adapted from previously developed critical appraisal tools for qualitative research studies [20,21]. The quality ratings were used to inform the quality-of-evidence assessment described below.

### Review management

Search results were uploaded to RefWorks (ThomsonResearchSoft, Philadelphia, PA), de-duplicated, and imported into the systematic review software DistillerSR (Evidence Partners, Ottawa, Canada) to conduct relevance screening, data extraction, and quality assessment. The relevance screening form was pre-tested before use on 50 abstracts to ensure consistent inclusion agreement (kappa value ≥0.8). The data extraction and quality assessment forms were also pre-tested on five articles prior to use to ensure questions were clear and consistently interpreted by both reviewers. Each stage of the review was conducted by two independent reviewers. Any reviewer conflicts were discussed to arrive at a consensus decision.

### Analysis

Qualitative analysis of all relevant articles was conducted using the thematic synthesis approach described by Thomas and Harden (2008). This approach was selected because the aim of the synthesis was to develop “analytical themes” that go beyond the primary studies to provide new insights and implications for policy, practice, and future research [16,18]. Both authors first independently reviewed each relevant article in detail and conducted line-by-line coding on the results sections [16]. This captured both “first-order” (participants’ interpretations of their experience) and “second-order” (authors’ interpretation of participants’ experience) concepts. We used an inductive approach to coding, without pre-formulated assumptions of how codes should be defined and structured. Codes were then iteratively compared and contrasted across studies to identify specific barriers and facilitators to safe food handling, which were grouped and organized under a set of descriptive themes [16]. We compared and discussed the barriers, facilitators, and descriptive themes across studies, and this process informed the development of analytical themes [16]. The analysis was conducted using PDFs of full articles imported into the NVivo 10 qualitative analysis software (QSR International, Doncaster, Australia).

The Confidence in the Evidence from Reviews of Qualitative research (CERQual) approach was adapted and used to assess how much confidence to place in each of the review findings [22]. CERQual is analogous to the Grades of Recommendation, Assessment, Development and Evaluation (GRADE) approach, which was developed by the Cochrane Collaboration to assess the confidence in evidence of effectiveness for outcomes in systematic reviews of interventions [23]. We applied CERQual to each identified barrier and facilitator in this review (sub-components of the descriptive themes). CERQual involves an assessment of four main criteria for each finding: 1) methodological limitations; 2) relevance; 3) coherence; and 4) adequacy of data [22]. An overview of each of these criteria as applied in this review is shown in Table 1. Based on the assessment for these four criteria, the overall confidence of each finding was then determined at one of three levels (modified from four in the original CERQual approach): high (it is highly likely that the review finding is a reasonable representation of the phenomenon of interest); moderate (it is likely that the review finding is a reasonable representation of the phenomenon of interest); and low (it is not clear whether the review findings are a reasonable representation of the phenomenon of interest). I.Y. conducted a preliminary CERQual assessment, which was reviewed and validated by L.W and finalized after discussion among both authors.

**Table 1:** Description of the CERQual approach used to assess confidence in each main review finding (adapted from [22]).

CERQual Components	Explanation	Rating options
Adequacy of data	Determination of the degree of richness and quantity of data supporting a review finding. Includes extent that data are supported by detailed narratives and participant quotes, and the number and diversity of studies represented.	No concerns Minor concerns Moderate concerns Substantial concerns
Relevance	The extent to which the body of evidence from the studies supporting the review finding is applicable to the review question context (population, phenomenon of interest, setting).	No concerns Minor concerns Moderate concerns Substantial concerns
Coherence	The extent to which the review finding is well grounded in data from the contributing studies and provides a convincing explanation for the patterns found in these data. Judgement based on consistency of data across studies, and ability to explain any contrasting or disconfirming data.	No concerns Minor concerns Moderate concerns Substantial concerns
Methodological limitations	The extent to which there are problems in the design or conduct of the contributing studies. Judgement based on each study’s relative contribution to the finding, the types of methodological limitations identified in the quality assessment tool, and how those limitations could impact on the specific finding.	No concerns Minor concerns Moderate concerns Substantial concerns
Overall assessment	Determination of overall confidence in review finding based on iterative review of each CERQual component.	High confidence Moderate confidence Low confidence

## Results

### Study characteristics

**Fig 1:**
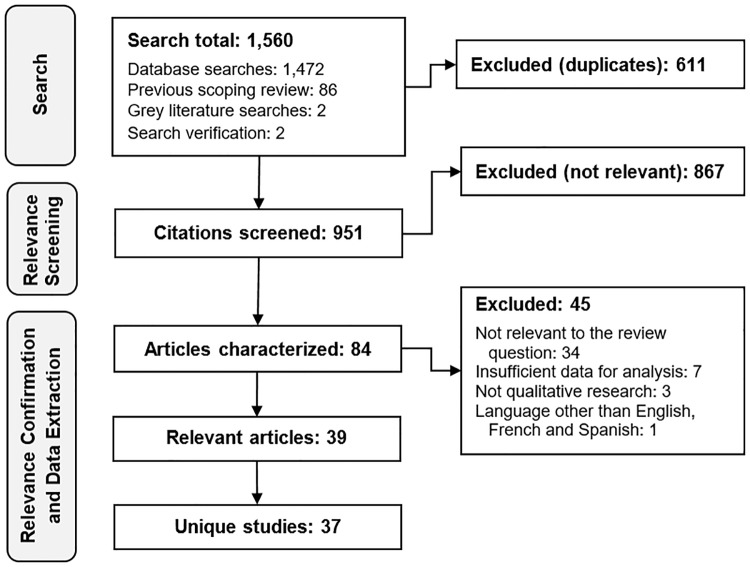
Systematic review flow chart.

A flow chart of the review process is shown in Fig 1. A total of 39 relevant articles were identified, reporting on 37 unique studies. A summary of the descriptive characteristics of these articles and studies is shown in Table 2. The median publication year of relevant articles was 2008 (range 1998–2015). Most of the 37 relevant studies (57%) were purely qualitative (vs. mixed-method), did not specify a guiding methodological or theoretical framework (60%), and used focus groups to collect qualitative data from participants (92%). Studies used a variety of recruitment methods and investigated several different target populations of consumers (Table 2). Among the 34 studies that conducted focus groups, the median number of groups conducted was 6 (range 1–12). The median total number of participants per study was 47 (range 8–96).

**Table 2:** Summary of the descriptive characteristics and quality assessment findings of 37 qualitative and mixed-method studies (39 articles) that investigated barriers and facilitators to consumer safe food handling.

Characteristics	No.	%
Document type of relevant article^a^:		
Journal article	31	79.5
Thesis	5	12.8
Government or research report	2	5.1
Conference paper	1	2.6
Study location^b^:		
USA	27	73.0
UK	6	16.2
Australia	1	2.7
Canada	1	2.7
Ireland	1	2.7
Italy	1	2.7
Switzerland	1	2.7
Study methodological/theoretical framework^b^:		
Health Belief Model	10	27.0
Ethnography	3	8.1
Extended Parallel Processing Model	1	2.7
Mental models approach	1	2.7
Phenomenology	1	2.7
Positive deviance approach	1	2.7
Protection Motivation Theory	1	2.7
Social marketing	1	2.7
Theory of Planned Behaviour	1	2.7
None stated	22	59.5
Qualitative data collection methods^b^:		
Focus groups	34	91.9
Interviews	6	16.2
Participant observation	3	8.1
Photo-elicitation and kitchen mapping	1	2.7
Participant recruitment methods^b^:		
Public notices (e.g. posters, flyers)	17	45.9
Public health, healthcare, or extension clinics / professionals	16	43.2
Community organizations, groups and centres	15	40.5
Market research firm or other database	8	21.6
Key informants / word of mouth	7	18.9
Not reported	3	8.1
Socio-demographic characteristics of targeted participants^b^:		
General population	11	29.7
Older adults / elderly	9	24.3
Racial / ethnic minorities (e.g. Hispanic, African American)	8	21.6
Pregnant / post-partum women	7	18.9
Parents / caregivers of young children	7	18.9
Low socio-economic status	4	10.8
Immuno-compromised individuals	4	10.8
College / university students	2	5.4
Focus of study topic^b^:		
General safe food handling behaviours	24	64.9
Safe handling of meat and poultry products	7	18.9
Listeriosis prevention	5	13.5
Use of food thermometers for cooking	3	8.1
Refrigeration practices	1	2.7
Quality assessment criteria:		
Clear statement of research aims	37	100.0
Research design and data collection strategy clearly described and appropriate to address the research aims	31	83.8
Sampling strategy clearly described and appropriate to address the research aims	33	89.2
Method of analysis clearly described and appropriate to address the research aims	14	37.8
Findings clearly described and supported by sufficient evidence	30	81.1
Evidence of researcher reflexivity	19	51.4
Ethical issues were taken into consideration	24	64.9
Evidence of study relevance and transferability	28	75.7

^a^ This question was tabulated out of 39 relevant articles. All other questions were tabulated out of the 37 total unique studies.

^b^ Multiple selections were possible for these questions.

A summary of the study quality assessment results is shown in Table 2. The most frequently deficient quality criteria included: not sufficiently describing the method of analysis (62%), not showing evidence of research reflexivity (49%), and not sufficiently describing or reporting ethical considerations (35%) (Table 2). Detailed study characteristics, quality assessment ratings, and a citation list of each relevant study are available as supplementary information (S1 Dataset and S3 File).

### Descriptive themes

Six descriptive themes were identified across the 37 relevant studies, describing a total of 31 different barriers and facilitators to safe food handling. These are described in detail below and are supported by illustrative quotes from participants in those studies (as reported by primary study authors). For brevity, quotes are only provided for findings rated as high or moderate confidence. Table 3 describes the CERQual confidence for each identified barrier and facilitator within the six descriptive themes. A more detailed table of the individual CERQual criteria ratings for each finding is available as supplementary information (S2 Table).

**Table 3:** Summary of the overall confidence in 31 barriers and facilitators to safe food handling across six descriptive themes, determined through the CERQual approach.

Theme / finding	Confidence in finding	Explanation of confidence rating
**Confidence and perceived risk**		
***Barriers***		
Lack of self-perceived risk due to confidence in own practices	High	Finding supported by 28 studies with rich data and minor methodological concerns
Belief in higher risk due to food prepared and handled by others	High	Finding supported by 21 studies with rich data, and minor methodological and coherence concerns
Not concerned about food safety because they have never previously experienced illness from food prepared at home	Moderate	Finding supported by 17 studies with limited data richness and moderate methodological concerns
Confidence in the food system to provide safe food	Moderate	Finding supported by 17 studies with minor adequacy of data, coherence and methodological concerns
Belief that foodborne illness is outside of consumers’ control	Low	Finding supported by only 5 studies with limited data richness
***Facilitators***		
Concern for dependents (e.g. children, elderly family members) at higher risk of foodborne illness and for whom they prepare food	High	Finding supported by 18 studies with rich data, and minor methodological concerns
Belonging to certain high-risk groups (e.g. immuno-compromised, first-time pregnant women) increases willingness to change food handling behaviours	Moderate	Finding is supported by 14 studies, with minor adequacy of data and methodological concerns, and moderate coherence concerns
Concern about the cost and inconvenience of foodborne illness	Moderate	Finding supported by 29 studies with rich data and minor methodological concerns, but some inconsistencies reported
Higher concern among those who have previously experienced foodborne illness or know someone who has, and believe it was due to food prepared at home	Low	Finding supported by 11 studies with minor methodological concerns, and moderate data richness and coherence concerns
**Knowledge-behaviour gap**		
***Barriers***		
Lack of knowledge and misconceptions about some recommended safe food handling practices	High	Finding supported by 34 studies with rich data, and minor methodological concerns
Disagreement with some recommendations for safe food handling due to conflicting beliefs and perceptions	High	Finding supported by 22 studies with minor coherence and methodological concerns
Some unsafe food handling behaviours followed despite being aware of recommended practices	Moderate	Finding supported by 13 studies with moderate data richness and minor methodological concerns
***Facilitators***		
Some recommended practices followed, often from the perspective of “common sense” and general hygiene than for food safety reasons	High	Finding supported by 32 studies with rich data, and minor methodological concerns
Willingness to learn more about food safety	Low	Finding supported by 15 studies with limited data richness and moderate concerns for relevance and coherence
**Habits and heuristics**		
***Barriers***		
Food handling behaviours are routine and unconscious, influenced by past experiences, and difficult to change	High	Finding supported by 29 studies with rich data, and minor methodological concerns
Various heuristics and “rules of thumb” used (e.g. sensory checks) when handling and preparing food	High	Finding supported by 31 studies with rich data, and minor methodological concerns
**Practical and lifestyle constraints**		
***Barriers***		
Inconvenience, lack of time, laziness and negligence contribute to unsafe practices	High	Finding supported by 25 studies with rich data, and minor methodological concerns
Distractions in the kitchen interfere with safe food handling	Moderate	Finding supported by 10 studies with limited data richness, and minor methodological concerns
Lack of proper resources and tools to facilitate safe food handling	Moderate	Finding supported by 19 studies with minor data richness, relevance, and methodological concerns
Inability to access or use resources due to kitchen layout or physical constraints	Low	Finding supported by only 5 studies with limited data richness and moderate methodological concerns
Safe food handling is another “burden” for some high-risk groups of consumers	Low	Finding supported by only 5 studies with limited data richness, limited applicability to other populations, and moderate methodological concerns
Reluctance to dispose of expired food among older adults	Low	Finding supported by only 5 studies with limited data richness, limited applicability to other populations, and moderate coherence concerns
Unique challenges for older adults and low-income households	Low	Finding supported by only 3 studies with limited data richness, limited applicability to other populations, and moderate coherence and methodological concerns
***Facilitators***		
Willingness to change behaviours if practical constraints were minimized or removed	Moderate	Finding supported by 12 studies with minor data richness and relevance concerns, and moderate methodological concerns
**Food preferences**		
***Barriers***		
Food choices driven by quality, perceived health benefits, and convenience over considerations for food safety	High	Finding supported by 15 studies with minor data richness and methodological concerns
***Facilitators***		
Preferred quality characteristics of safely prepared foods	Low	Finding supported by 10 studies with limited data richness and moderate methodological concerns
**Societal and social influences**		
***Barriers***		
Cultural traditions associated with some unsafe food handling practices	Moderate	Finding supported by 9 studies with limited data richness and minor methodological concerns, but with diversity of populations represented
Unsafe practices learned through family, friends, and social networks	Moderate	Finding supported by 13 studies with minor data richness concerns and moderate methodological concerns
Negative social acceptability of some recommended practices	Low	Finding supported by 7 studies with limited richness in data, and some relevance, coherence, and methodological concerns
***Facilitators***		
Healthcare providers and extension services as trusted sources of food safety information	High	Finding supported by 18 studies with rich data, and minor methodological and coherence concerns
Media stories and coverage increase food safety awareness	Moderate	Finding supported by 16 studies with rich data and minor methodological concerns, but with moderate coherence concerns

### Confidence and perceived risk

We identified high confidence in two barriers and one facilitator under this theme (Table 3). Firstly, consumers generally did not perceive that they were at risk of contracting foodborne illness due to food prepared at home. They tended to express confidence in their own ability to handle and prepare food safely at home, and did not believe that foodborne illness was likely to happen to them or that the consequences were severe enough to warrant changing their behaviours.

“I’m not going to, or have no intention of changing my ways right now. And I feel very confident in what I do cook, and that I do it correctly, so I don’t feel the need for it [thermometer to check doneness of meat].” [24]

Secondly, consumers were generally more concerned about the safety of foods prepared and handled by others and in external settings (e.g. grocery stores, restaurants) than food they handled and prepared at home.

“The only thing you can control is what you have in your own house.” [25]

Thirdly, many consumers indicated that they were more likely to practice safe food handling behaviors when preparing food for others, such as children or other susceptible populations (e.g. the elderly, immuno-compromised individuals).

“I pay more attention to the way I do things because I now have children. It gives me a totally different perspective.” [26]

Moderate confidence was found for two barriers and two facilitators under this theme (Table 3). Consumers often rationalized that they have always practiced a particular unsafe handling behavior and have never been sick as a reason not to change their practices, or for not being concerned about food safety.

“I am 66 years old, and it (food poisoning) hasn’t happened yet.” [27]

Some consumers expressed confidence and trust in the food system to supply safe food for distribution and purchase, decreasing their concern and belief in the need for additional food safety precautions at home.

“I figure it’s okay or they wouldn’t be allowed to sell it.” [28]

Consumers experiencing a change in health status, such as the immuno-compromised and first-time pregnant women, tended to be more concerned about contracting foodborne illness and taking some precautions at home to prevent it.

“If it’s not safe, then I will not eat it for the rest of my pregnancy then.” [29]

Consumers in some studies indicated that increased awareness and understanding of the negative and severe consequences of foodborne illness (e.g. costs, inconvenience) would motivate them to practice safer food handling behaviours at home.

“I’m a single parent so I would just have to cancel the whole day and just stay home with them.” [30]

We identified low confidence in two findings under this theme: that consumers in some studies believed foodborne illness was more a matter of “bad luck” and chance than something under their control; and that some consumers who had previously contracted foodborne illness or knew someone else who had become ill were more motivated to handle food safely at home. However, for the latter finding, some consumers tended to attribute any previous illness to food eaten outside of the home (e.g. at a restaurant), or would be more likely to stop eating the implicated food or product brand name than to change their practices.

### Knowledge-behaviour gap

Three of the five barriers and facilitators identified under this theme were rated as high confidence (Table 3). Consumers were knowledgeable about the general concepts of many recommended safe food handling practices (e.g. sanitation and cleanliness). These were followed to various degrees, often from the perspective of “common sense” and for general hygiene purposes, rather than explicitly as a food safety practice.

“Make sure you wash hands, frequently… or much as you can, it doesn’t matter how many times.” [31]

Some frequently identified areas of lack of knowledge and awareness were also identified (e.g. proper refrigeration temperatures, not washing chicken before cooking it, some risky foods that should be avoided by high-risk groups). In some cases, participants often believed that they were following the correct practice.

“I didn’t know that about 2 hours. We leave it on the stove as we eat and as night falls and everyone is going to bed, then you put it away.” [32]

Consumers often disagreed with or were skeptical of recommended safe food handling practices (e.g. storage and reheating of leftover foods, using thermometers to check the doneness of meat) because these conflicted with their prior beliefs and misconceptions.

“It’s all a load of rubbish I think… some people say you shouldn’t heat meat more than twice, re-heat it, but I’ve done it three or four times I’m still here, I’m fine.” [33]

Moderate confidence was found in the finding that knowledge did not always correspond to actual or self-reported use of safe food handling practices. Consumers often acknowledged engaging in unsafe behaviors despite being aware of the recommended practice.

“I know you shouldn’t, but I prefer meat to be left to breathe. If I’m cooking a joint, it sits out for eight hours, you know, an unhealthy time.” [34]

We identified low confidence in one finding under this theme: that some consumers, particularly those in high-risk groups and young adults, were more likely to indicate a willingness to learn more about safe food handling practices.

### Habits and heuristics

Both findings identified under this theme were rated as high confidence (Table 3). Across the analyzed studies, consumers’ food handling behaviours were described as routine and habitual, occurring largely as an unconscious activity that was influenced by their past experiences and how they learned to cook growing up.

“I’ve been cooking all of my life so I pretty much know… I know how to size my food up, when it is done and when it is not done.” [35]

Consumers frequently used various heuristics and “rules of thumb” when handling and preparing food, guided by their intuition, trial-and-error and “common sense”. Sensory judgements (e.g. visual checks, smelling, tasting, and feeling the texture of foods) were frequently used to evaluate cooking doneness and to determine whether food was spoiled.

“I’m a big fan of hacking it open and looking inside. That’s tried and true.” [24]

“I wiggle the turkey leg. If it’s loose, I guess it’s done.” [36]

### Practical and lifestyle constraints

One finding identified under this theme was rated as high confidence (Table 3). Several safe food handling practices were often seen as inconvenient (e.g. using a thermometer to check meat doneness, using separate cutting boards for raw and ready-to-eat foods, reheating deli meats for high-risk groups) or impractical in some situations (e.g. using a thermometer when cooking thin cuts of meat). Consumers often noted a lack of time, laziness, “hassle” and negligence as contributing barriers.

“My schedule is very tight in the evening. I come back home at five, and take care of Alice (granddaughter), and in the same time cook, and eat… you know, during that time, washing hands for 20s or checking the cooking temperature is just difficult.” [29]

Three findings under this theme were rated as moderate confidence (Table 3). A lack of necessary tools (e.g. thermometer, separate cutting boards) and appliances (e.g. dishwasher for easier sanitation of utensils, large enough freezer or refrigerator for storage) was frequently noted as a barrier. A lack of finances to purchase these resources was commonly mentioned as a contributing factor.

“That thermometer looks like it‘s probably expensive. That would keep me from using it.” [37]

Consumers in several studies noted that they would be more willing to change their behaviours if practical constraints (e.g. inconveniences, lack of tools) were removed or minimized, such as if they were to receive a free thermometer or if they could save time.

“Maybe if it’s some sort of time saver, in that you realize that you reach a certain temperature in a shorter time than you think. So, you don’t have to cook the food as long as you thought. It’s actually ready before you thought.” [24]

Distractions in the kitchen, such as children, pets, other activities or having multiple people in the house were also sometimes viewed as barrier.

“My granddaughters know how to get in the fridge, they’ll get food out and I'll find it later… it's really bad.” [30]

The following four additional barriers were rated as low confidence under this theme: inadequate kitchen layout and difficulty in accessing necessary resources (e.g. having to dig a thermometer out of drawer); the health status of some high-risk individuals (e.g. immuno-compromised, pregnant women), in that their diet is already restricted or they have to deal with other health issues, and food safety is an added inconvenience; a reluctance to dispose of expired or spoiled food among older adults (e.g. due to thriftiness or experiences with food shortages during childhood); and for some low-income households and older adults, a lack of ability to access reliable transportation to the grocery store, and long travel distances to the grocery store, which could have food safety consequences.

### Food preferences

High confidence was identified for the finding that consumers tended to make food choices based on quality characteristics, perceived health benefits and convenience rather than for food safety reasons. For example, many studies indicated that consumers prefer the taste, texture, and appearance of some unsafely prepared or risky foods (e.g. raw and undercooked eggs and egg products, undercooked meat).

“I do not consider over-easy raw because that is how I like my eggs” [38]

Low confidence was identified for the finding that some consumers prefer the taste of safely prepared foods (e.g. meat no longer overcooked due to using a thermometer to confirm doneness).

### Societal and social influences

High confidence was identified for the finding that consumers trust and prefer to receive information about food safety from healthcare professionals and extension services. However, despite being a trusted source, some studies of pregnant women suggested that their doctors did not always provide sufficient food safety advice.

“I would probably speak to my doctor or midwife because they’re the sort of people that deal with this stuff every day.” [39]

Moderate confidence was identified for three findings under this theme (Table 3). Media stories and in-depth coverage about food safety (e.g. recalls, outbreaks) were noted to have some, at least short-term, influence on consumers’ awareness of food safety issues. However, some consumers were skeptical of media “hype” and “scare tactics”.

“It pricks your memory all the time, it makes you aware if you eat something.” [31]

Some consumers in different ethnic groups reported potentially unsafe food handling practices influenced by their cultural traditions (e.g. Asian and Hispanic consumers purchasing of live poultry in urban markets which are then slaughtered and brought home, and Hispanic consumers eating of ‘queso fresco’ soft cheese which is traditionally made from unpasteurized milk).

“Whenever I visit my mother in Mexico, I bring home-made cheese prepared with milk from her cows.” [40]

Some consumers noted that they learned certain unsafe food handling practices and habits from their family (e.g. parents), friends, and social networks.

“My mother puts meat in a bowl with water on the counter for the day.” [41]

One finding under this theme was rated as low confidence: some safe food handling practices (e.g. using a food thermometer) were viewed as socially unacceptable or not socially desirable in some situations (e.g. parties, barbeques).

### Analytical themes

We identified three overarching analytical themes that describe the implications of the above findings. Each is discussed in sequence below.

### Safe food handling behaviours occur as part of a complex interaction of everyday consumer practices and habituation

This theme reflects how consumers’ use of safe food handling behaviours, or lack of, was typically driven by unconscious actions rooted in habit and routine, rather than through deliberate action and rational logic. Consumers tended only to actively think about and take extra food safety precautions under special or unfamiliar circumstances (e.g. preparing large meal for a family gathering, preparing a food for the first time). Within the context of their everyday food handling situations, consumers were generally confident in their abilities, and this was a strong determinant of their willingness to change their behaviours. This confidence typically increased with age and experience, thus younger adults were more willing to try to adopt safe food handling recommendations into their food preparation routine.

### Most consumers are not concerned about food safety and are generally not motivated to change their behaviours based on new knowledge about food safety risks

Adequate knowledge of food safety risks and safe food handling practices is necessary but not sufficient to change consumer behaviours. Even when safe food handling practices were being conducted by consumers (e.g. cleaning of kitchen surfaces, proper organization of refrigerator), this was often not done with food safety explicitly in mind, but for general cleanliness purposes, for convenience, or because of food preferences. While some consumers indicated that they would like to learn more about food safety, there were mixed reactions regarding whether this information would be adopted into practice. One notable exception was that individuals who were experiencing a change in health status (e.g. first-time pregnant women, immuno-compromised) showed a stronger willingness to actively seek out food safety information and to modify at least some their behaviours accordingly.

### Consumers are amenable to changing their safe food handling habits through relevant social pressures

Consumers often expressed concern for their children and other dependants (e.g. the elderly), and indicated that they would be more likely to change their practices when preparing food for them. Parents of young children also expressed how they would be influenced by what their kids learn in school:

“When it comes home from the school it’s like, ‘Hey, we’ve got to read this. It’s a warning.’ They’re telling us about something we need to know for our kids.” [42]

Healthcare professionals and extension services were seen as credible sources of food safety information that consumers tend to look to for advice, and some consumers were also influenced by the media, cultural traditions, their family and friends, and to a lesser extent (particularly for food thermometer usage), the social acceptability of the practice.

## Discussion

We identified and synthesized data from a diverse body of primary qualitative research literature on the barriers and facilitators to safe food handling among consumers. However, most of the research was conducted in the US, and it is possible that some barriers and facilitators could differ by country or geographic region. There is a need to conduct additional qualitative research of the underlying reasons affecting consumers’ safe food handling behaviours in other countries, particularly in targeted sub-populations that are at higher risk of foodborne illness and its severe consequences (e.g. older adults, immuno-compromised individuals, pregnant women).

While most recommended quality criteria were met by the included studies, some deficiencies were identified (Table 2). In particular, most studies did not sufficiently describe their method of analysis (e.g. only reporting that themes “emerged” from the data but without providing a clear account of the actual steps taken to achieve this), which makes it difficult to judge whether the findings are adequately supported by the data [43]. Half of the studies did not provide any evidence of research reflexivity, which refers to documentation of how the researchers potentially shaped the data collection and interpretation process, including the role of any prior assumptions and experience [43]. This is important to allow the reader to make judgements on any potential biases and the credibility of the findings [20,43]. Finally, several studies did not provide any explicit indication that their study had been reviewed and approved by an institutional research ethics board, or that ethical standards were taken into account (e.g. through participant consent and provision of confidentially). Future researchers of qualitative studies in this area should follow recommended reporting guidelines for qualitative research studies, such as the Standards for Reporting Qualitative Research (SRQR) or the consolidated criteria for reporting qualitative research (COREQ) [44,45].

We found that consumers’ food handling behaviours were largely influenced by routine, unconscious actions and experiential knowledge. Social theories of practice provide a framework to explain safe food handling behaviours from this perspective [46], and they have recently been applied to investigate and explain the safe food handling behaviours of consumers in the United Kingdom [33,34,47–49]. Understanding consumer food handling behaviour within this context could be used to guide the development of future interventions. For example, one approach to increasing consumer adoption of safe food handling behaviours could be to instill new habits and routines by creating a new “norm”. This can be accomplished by putting people in unfamiliar situation (e.g. outside of their home) for hands-on training, to learn and practice new techniques and to do things differently [46]. The Expanded Food and Nutrition Education Program (EFNEP) in the US is an example of a current food safety and nutrition education initiative that uses such an approach. Training is conducted by paraprofessionals (i.e. peer educators) in community settings (e.g. centers, schools, places of worship) and is targeted at low-income families [50]. Previous studies have shown that this experiential, peer-learning approach is effective to improve safe food handling behaviours [51,52].

Another approach to overcoming consumers’ habitual food handling practices could be to target young adults and children, who are still developing their habits and have little experiential knowledge. Several interventions for school-aged children and college students have been shown to be effective at improving food safety knowledge, attitudes and behaviours in previous studies [53], including innovative and engaging approaches such as interactive games, music parodies, and social media [54–56]. Young adults were identified as consistently indicating a willingness to learn and change their behaviours. Targeting school-aged children may also influence their parents’ practices at home, and thus could have a wider impact. Consumers undergoing a change in lifestyle either due to a health event or a change in their family or living situation may be a good target for intervention (e.g. first-time pregnant women, families caring for an immuno-compromised individual), to capitalize on their increased willingness to learn about food safety and modify their practices during this time.

We found that consumers were generally confident about their safe food handling practices at home, even though they lacked knowledge in some areas and had some misconceptions. For example, consumers frequently used sensory evaluations (e.g. visual appearance, smelling) to check cooking doneness and spoilage, and these practices are known to be unreliable indicators of microbial food safety [57,58]. We also found that consumers were generally not concerned about food safety and frequently engaged in unsafe food handling behaviours even when they were aware of the recommended practice, which corresponds with the findings of previous quantitative surveys [59–62]. As a result, interventions that rely on passive communication of facts and information about food safety risks are unlikely to result in sustainable consumer behaviour change. Education campaigns, messages, and interventions should incorporate other aspects that tend to motivate consumers (e.g. quality enhancements, convenience) [24,27,42,63]. For example, safe internal cooking temperatures could be included in cookbooks and online recipe websites to overcome consumers’ lack of knowledge of safe food temperatures and the inconvenience of looking them up. The most successful interventions will likely be those that take a multi-faceted approach and include sustained efforts over time to achieve more effective behaviour change [53].

Social and cultural influences are important factors affecting consumers’ use of safe food handling behaviours. To promote behaviour changes that challenge existing social or cultural norms, interventions may need to appeal to consumers’ concerns for their children and other dependants, or carefully suggest changes that appear to be in line with cultural norms. One example is “photonovella”, a text- and visual-based storytelling format that has been shown in recent studies to positively affect consumers’ food safety behaviours [35,64]. More comprehensive approaches that use radio, television, or multi-platform programs to promote behaviour change through fictional melodramatic narratives, known as entertainment–education or “edutainment” programs, have been effectively used in many different global settings to address various health behaviours [65], and could have potential applications to the area of consumer food safety. Healthcare professionals (e.g. doctors, midwives) and extension service clinics, such as the Special Supplemental Nutrition Program for Women, Infants, and Children (WIC) in the US, were viewed as credible sources of information, and should be engaged as a first point of contact for providing essential food safety information to high-risk consumers and referring them to local public health resources for additional information, education, and training programs (e.g. EFNEP in the US).

Above we noted theories of practice as a framework to explain consumer safe food handling behaviours; other studies have investigated the role of different socio-cognitive theories for this purpose. One of the most commonly investigated socio-cognitive theories within this context is the Theory of Planned Behaviour, which suggests that an individual’s behaviour is mediated by their intentions to perform the behaviour, and that their intentions can be predicted by their attitudes toward the behaviour, their subjective norms (i.e. social pressure to perform the behaviour), and their perceived behavioural control (i.e. perceived ability to perform the behaviour) [66]. Several recent quantitative studies have found that the Theory of Planned Behaviour and its constructs (in particular, subjective norms and perceived behavioural control) are associated with safe food handling behavioural intentions and self-reported behaviours among consumers [67–71]. Although only one of qualitative studies captured in this review was explicitly guided by this theory, we found that there is an underlying psychosocial basis for each of the Theory of Planned Behaviour constructs, and these appear to be important factors affecting consumer safe food handling behaviours. As a result, this theory may provide a good foundation from which future interventions could be constructed so that the most important and appropriate constructs can be targeted for behaviour change in specific populations and contexts.

Qualitative research studies can be difficult to identify in bibliographic databases due to widely variable indexing practices [16,72]. Therefore, it is possible that we could have missed some potentially relevant articles in this review. However, we are confident that we achieved conceptual saturation of the main findings, given the diverse number of included articles, and believe that the inclusion of any additional articles in the analysis would not change the key themes, interpretations, or conclusions. The CERQual approach to assessing confidence in the review findings involved some subjectivity. However, we attempted to minimize any potential biases in this approach by pre-specifying standardized criteria, and by having two reviewers complete the process. We reduced the original number of confidence levels from four to three, as we found it challenging to make confident assessments beyond three levels of stratification. At the time of application, the CERQual approach was still in development [22]. However, we nevertheless felt that it added value to the interpretation of the findings and improved the usability of results for decision-making.

## Conclusions

We used structured and transparent knowledge synthesis methods to synthesize qualitative data from primary research studies on the barriers and facilitators to safe food handling among consumers. Overall, we found that consumer safe food handling behaviours are implemented largely as unconscious, habitual and routine activities for which they tend to have strong confidence in their abilities; that consumers are generally not concerned about food safety risks; and that increased knowledge alone is unlikely to change their behaviours. It was noted that certain, relevant social pressures (e.g. children) and credible sources (e.g. healthcare professionals) provide potentially influential opportunities to impact behaviour change. Social theories of practice and the Theory of Planned Behaviour provide frameworks that can be used to investigate these constructs further in primary research and to design future intervention strategies. Potentially promising educational strategies include one or more features: hands-on training in community settings; targeting young adults, children, and individuals experiencing a change in lifestyle or health status; emphasizing motivating factors such as quality enhancements and ability to save time; and use of engaging and multi-faceted techniques. Future primary research using robust study designs is warranted to investigate and evaluate such strategies in different contexts and settings, particularly for high-risk population groups.

## Supporting Information

S1 File Systematic review protocol and forms. (DOCX)

S2 File Additional search strategy details. (DOCX)

S3 File Citation list of relevant articles. (DOCX)

S1 Table ENTREQ checklist. (DOCX)

S2 Table Detailed CERQual ratings. (DOCX)

S1 Dataset Detailed study characteristics. (XLSX)
