# Supplementary material for: Barriers and Facilitators to Safe Food Handling among Consumers: A Systematic Review and Thematic Synthesis of Qualitative Research Studies
Source: PLoS One. 2016 Dec 1;11(12):e0167695. doi: 10.1371/journal.pone.0167695 (PMC5132243; doi:10.1371/journal.pone.0167695)
Supplement: S1 File — (DOCX) [file pone.0167695.s001.docx]

S1 File: Systematic Review Protocol and Forms

**Review rationale and approach:**

This review aims to provide a deeper understanding of factors related to safe food handling among consumers in developed countries. Numerous primary qualitative research studies have been conducted to investigate such factors in different consumer populations and settings. To date no transparent and structured approach has been used to integrate the insights from these studies.

This protocol proposes an approach for conducting a systematic review using thematic synthesis to integrate evidence and obtain new insights from primary qualitative research studies on barriers and facilitators to safe food handling among consumers in developed countries (Barnett-Page and Thomas, 2009; Thomas and Harden, 2008; Tong et al., 2012). It is expected that the results can be used to inform future research, policy and practice related to the development of food safety education and behaviour-change interventions for consumers.

**Review question:**

The review question is as follows: “What are the barriers and facilitators to safe food handling among consumers in developed countries?” Eligibility criteria are outlined in the table below:

| Parameters | Inclusion criteria | Exclusion criteria |
| --- | --- | --- |
| Participants | Research on adult consumers (≥18 years old) who prepare or handle food for consumption at home | Research on food handlers employed in the food industry, educators of consumers, and children and youth (<18 years old) |
| Topic | Research on barriers and/or facilitators related to one or more of the following ***microbial food safety constructs***: personal hygiene; avoiding cross-contamination; adequate cooking; keeping food at safe temperatures; and avoiding “risky” foods (Medeiros et al., 2001) | Research related to chemical and other hazards (e.g. GMOs) |
| Location | Research conducted in developed countries/regions (classified as “very high human development” by the United Nations Development Programme) |  |
| Study types | Any qualitative or mixed-method primary research design published in English, French, or Spanish | Non-primary qualitative research, quantitative research |

**Updated search:**

All relevant qualitative studies (n=86) identified in a previous scoping review about food safety education interventions for consumers (Sivaramalingam et al., 2015) will be considered for analysis. In addition, an updated search will be conducted in the same bibliographic databases using a modified version of the search algorithm as the previous scoping review. The original algorithm included a combination of food safety-related terms (e.g. food safety), population terms (e.g. consumer, client, adults), intervention terms (e.g. program, course, campaign), and outcome terms (e.g. behavior, knowledge, attitudes). In the updated search, the category of “intervention” terms will be removed, and another category of “qualitative research” terms (qualitative OR “focus group*”) will be added to focus the search on relevant research designs, and some population key words will be removed according to the eligibility criteria. The search will be re-run for all years to identify any additional studies that might not have been captured with the original intervention terms included.

**Relevant screening and utility assessment:**

The titles and abstracts of all identified articles will be screened using a pre-specified form of one question to determine whether the study meets the core eligibility criteria (Form 1). Full papers of relevant articles will be obtained and confirmed for relevance. Depending on the number of relevant studies identified, more detailed thematic synthesis may be further prioritized based on: geographic location of the study, key target population/participants and their characteristics, and/or topics covered.

**Data extraction and quality assessment:**

A pre-specified form will be used to extract key characteristics from the articles: study aim and location, type and number of participants, and methodology used (Form 2). All relevant studies will then be critically appraised using a quality assessment form containing eight questions (Form 3). The criteria were adapted from previously developed critical appraisal tools for qualitative research studies (Critical Appraisal Skills Programme, 2013; Walsh and Downe, 2006). Results of the assessment will not be used to exclude studies lacking in key criteria, but will be used in the assessment of confidence in the findings.

**Analysis:**

Analysis will be conducted using the thematic synthesis approach described by Thomas and Harden (2008). Two independent reviewers will conduct all steps. Firstly, each relevant article will be read in detail and “line-by-line” open (inductive) coding will be conducted on the results sections (Thomas and Harden, 2008). Codes will then be iteratively compared and contrasted across studies to develop descriptive themes (Thomas and Harden, 2008). These will be compared across reviewers, discussed, and then used to develop “analytical themes”. The analytical themes aim to “go beyond” the original data by comparing descriptive themes across studies to provide new insights related to the review question (barriers/facilitators to safe food handling) (Thomas and Harden, 2008). The analysis will be conducted using qualitative analysis software (e.g. NVivo).

The Confidence in the Evidence from Reviews of Qualitative research (CERQual) approach, developed analogous to the Grades of Recommendation, Assessment, Development and Evaluation (GRADE) approach for qualitative syntheses (Guyatt et al., 2011), will be used to assess how much confidence to place in each of the individual findings of the review (Lewin et al., 2015). Specifically, the CERQual assessment will be conducted for each main analytical theme. CERQual involves an assessment of four main criteria for each finding: 1) methodological limitations; 2) relevance; 3) coherence; and 4) adequacy of data (Lewin et al., 2015). Based on the assessment for these four criteria, the overall confidence of each finding is then determined at one of four levels: high; moderate; low; or very low. As the CERQual approach is still being developed, it will be adapted to suit the particular needs and context of this review.

**Reporting of results:**

The review will be reported in accordance with the ‘Enhancing Transparency in Reporting the Synthesis of Qualitative Research’ (ENTREQ) reporting guidelines (Tong et al., 2012).

**Protocol References:**

Barnett-Page, E., Thomas, J., 2009. Methods for the synthesis of qualitative research: a critical review. BMC Med. Res. Methodol. 9, 59.

Critical Appraisal Skills Programme, 2013. 10 questions to help you make sense of qualitative research. Available at: <http://media.wix.com/ugd/dded87_29c5b002d99342f788c6ac670e49f274.pdf>.

Guyatt, G., Oxman, A.D., Akl, E.A., Kunz, R., Vist, G., Brozek, J., Norris, S., Falck-Ytter, Y., Glasziou, P., Debeer, H., Jaeschke, R., Rind, D., Meerpohl, J., Dahm, P., Schünemann, H.J., 2011. GRADE guidelines: 1. Introduction - GRADE evidence profiles and summary of findings tables. J. Clin. Epidemiol. 64, 383–394.

Lewin, S., Glenton, C., Munthe-Kaas, H., Carlsen, B., Colvin, C.J., Gülmezoglu, M., Noyes, J., Booth, A., Garside, R., Rashidian, A., 2015. Using Qualitative Evidence in Decision Making for Health and Social Interventions: An Approach to Assess Confidence in Findings from Qualitative Evidence Syntheses (GRADE-CERQual). PLOS Med. 12, e1001895.

Medeiros, L., Hillers, V., Kendall, P., Mason, A., 2001. Evaluation of food safety education for consumers. J. Nutr. Educ. 33 Suppl 1, S27–34.

Sivaramalingam, B., Young, I., Pham, M.T., Waddell, L., Greig, J., Mascarenhas, M., Papadopoulos, A., 2015. Scoping review of research on the effectiveness of food-safety education interventions directed at consumers. Foodborne Pathog. Dis. 12, 561–570.

Thomas, J., Harden, A., 2008. Methods for the thematic synthesis of qualitative research in systematic reviews. BMC Med. Res. Methodol. 8, 45.

Tong, A., Flemming, K., McInnes, E., Oliver, S., Craig, J., 2012. Enhancing transparency in reporting the synthesis of qualitative research: ENTREQ. BMC Med. Res. Methodol. 12, 181.

Walsh, D., Downe, S., 2006. Appraising the quality of qualitative research. Midwifery 22, 108–119.

***Form 1: Relevance Screening Form***

| **Question** | **Options** | **Definitions/additional notes** |
| --- | --- | --- |
| 1. Does the citation describe primary qualitative or mixed-method research investigating barriers and/or facilitators to safe food handling among adult consumers in developed countries? | - Yes - No | Food safety:  Practicing safe handling, preparing, storing, and consumption of food in order to prevent foodborne illness (e.g. personal hygiene and hand-washing, avoiding cross contamination, adequate cooking of food, time-temperature control, avoiding food from unsafe sources). Includes microbial, chemical and/or physical hazards that could cause foodborne illness or other adverse health effect.  Consumers:  The population of interest is adult (≥18 years old) consumers, including the general public, home cooks, household food preparers, high risk populations (e.g. immune compromised, pregnant women, elderly), ethnic groups, and volunteer cooks for special food events or bake sales hosted by religious organizations, service clubs or fraternal organizations.  Include   - All study designs, publication dates, and publication types, including grey literature (e.g. public opinion research) - Studies published in English, French or Spanish - Studies where you “can’t tell” the relevance and suspect it could be relevant   Exclude   - Studies *not directly related to food safety* (e.g. generic hand-washing only). Studies on water quality/safety should also be excluded unless reported in a food safety context (e.g. safe water for food preparation). - Research on GMOs and food allergies. - Studies focusing on *food handlers* employed in the food industry and *educators of consumers* |

***Form 2: Relevance Confirmation and Data Extraction Form***

| **Question** | **Options** | **Comments** |
| --- | --- | --- |
| 1) Does the citation describe primary qualitative or mixed-method research published in English, French, or Spanish investigating barriers and/or facilitators to safe food handling among adult consumers in developed countries? | Yes  No, specify reason(s) for exclusion:   - Not relevant to review question: ____________ - Insufficient data for analysis:_____________ - Other:_______________   *If “no” is selected, do not proceed further.* | **Consumers:** The population of interest is consumers, including the general public, home cooks, high risk populations (e.g. immune compromised, pregnant women, elderly), ethnic groups, and volunteer cooks for special food events or bake sales hosted by religious organizations, service clubs or fraternal organizations. Research on ***food handlers*** and ***educators of consumers (e.g. health providers)*** should be **excluded**.  **Primary research**: a study where the authors collected and analyzed their own data – and report original results (including participant quotes).  **Developed countries:** includes any of the countries with “very high human development” in the UN Development Programme’s Human Development Report: <http://hdr.undp.org/en/countries>  **Insufficient data:** qualitative not detailed enough to analyze for themes (e.g. only a few sentences or very brief section of results is given, and/or limited to no illustrative participant quotes are provided).  **Not relevant:** the following should be excluded based on irrelevance to the review question:   - Studies that only measure participant response/evaluation to communication or education materials without exploring barriers/facilitators to safe food handling - Studies that do not investigate safe food handling in the context of the following **microbial food safety** constructs: personal hygiene; avoiding cross-contamination; adequate cooking; keeping food at safe temperatures; and avoiding “risky” foods. - Studies that measure consumer responses to in outbreak/emergency situations. |
| 2) What is the publication year of this article? | _______ | Enter year |
| 3) What type of document is this article? | Journal article  Conference proceedings  Government or research report  Thesis  Book or book chapter  Other, please specify:_____ |  |
| 4) What is the article language? | English  French  Spanish |  |
| 5) Where was the study conducted | _________ | **Enter country, state/province** |
| 6) What is the study methodology? | _________ | Example designs include descriptive, grounded theory, phenomenology, ethnography, or mixed-method. |
| 7) How were data collected? | Qualitative interviews:___________  Focus groups:__________________  Participant observation: ____  Analysis of documents: ____  Questionnaire:_____  Other: ______ | Specify details of data collection |
| 8) How were participants recruited? | ___________ |  |
| 9) When was the study conducted? | ___________  Not reported | Please specify year/month to year/month if available (do not extract days) [follow format example: 2000/06-2000/08] |
| 10) What were the key socio-demographic characteristics of the *participants*? | ___________ | Specify gender, age range, race/ethnicity, vulnerabilities (e.g. pregnant women, immune-compromised) or other specifics of participants investigated. |
| 11) What was the aim of the study as specified by the authors? | ___________ |  |
| 12) Additional comments: | __________ |  |

***Form 3: Quality Assessment Form***

| **Question/quality domain** | **Options** | **Definitions/additional notes** |
| --- | --- | --- |
| Was there a clear statement of the research purpose/aims? | - Yes - No | Consider the following to make a judgement:   - Clarity of focus, explicit purpose given, supported by prior research |
| Was the research design and data collection strategy clearly described and appropriate to address the research aims? | - Yes - No | Consider the following to make a judgement:   - Rationale provided for research design/data collection strategy (including setting) - Research design and data collection strategy were appropriate to address the research purpose/question |
| Was the sampling strategy clearly described and appropriate to address the research aims? | - Yes - No | Consider the following to make a judgement:   - Selection criteria detailed, and description provided for how sampling was undertaken - Justification for sampling strategy and selection of participants is given. |
| Was the method of analysis clearly described and appropriate to address the research aims? | - Yes - No | Consider the following to make a judgement:   - Approach made explicit (e.g. thematic analysis, grounded theory) and described in depth - Discussion of how coding systems/conceptual frameworks evolved - If thematic analysis conducted, is it clear how themes were derived? |
| Were the findings clearly described and supported by sufficient evidence? | - Yes - No | Consider the following to make a judgement:   - Did data provide sufficient depth, detail and richness? (e.g. illustrative quotes) - Context described and taken into account in interpretation/results - Approaches taken to ensure robustness (e.g. multiple analysts, triangulation, member checking/participant validation of results) |
| Was there evidence of researcher reflexivity? | - Yes - No | Consider the following to make a judgement:   - Discussion of relationship between the researchers and participants during data collection - Researchers’ potential role and influence on study critically examined and/or discussed - Evidence of how problems/complications met were dealt with |
| Were ethical issues taken into consideration? | - Yes - No | Consider the following to make a judgement:   - Study approved by ethics committee - Sufficient details provided on how the research was explained to participants and whether ethical standards were maintained - Documentation of how autonomy, consent, confidentiality, anonymity were managed - Documentation of any ethical dilemmas and how they were resolved |
| Was there evidence of study relevance and transferability? | - Yes - No | Consider the following to make a judgement:   - Discussion of contribution of study to existing/prior knowledge, practice, and/or policy - Areas for future research identified - Limitations/weaknesses of study clearly outlined - Discussion of whether or how the findings can be transferred to other populations or consideration of other ways the research may be used |
