# Supplementary material for: Barriers and Facilitators to Safe Food Handling among Consumers: A Systematic Review and Thematic Synthesis of Qualitative Research Studies
Source: PLoS One. 2016 Dec 1;11(12):e0167695. doi: 10.1371/journal.pone.0167695 (PMC5132243; doi:10.1371/journal.pone.0167695)
Supplement: S2 File — (DOCX) [file pone.0167695.s002.docx]

S1 File – Additional Search Details

Detailed Search Algorithms for Bibliographic Database Searches

| **Date** | January 8, 2016 |
| --- | --- |
| **Platform/Interface** | OvidSP |
| **Databases** | CAB Abstracts (1973-2016)  Agricola (1970-May 2016)  Food Science and Technology Abstracts (1969-2016)  PsycINFO (1806-2016) |
| **Institution** | Public Health Agency of Canada |
| **Search string 1:** | *((("food safety" OR "food-borne" OR foodborne OR foodbourne OR “food-bourne” OR "food handling" OR "food preparation" OR "food poisoning" OR “food hygiene” OR “safe food”) AND (consumer* OR client* OR population OR public OR people OR person OR persons OR individual OR individuals OR student OR students OR mother* OR father* OR adult OR adults OR women OR female* OR communit* OR families OR family OR household* OR domestic OR volunteer OR volunteers OR home OR homes OR campus OR university OR universities OR college*) AND (awareness OR knowledge OR practice* OR behaviour* OR behavior* OR attitude* OR perception* OR preference* OR learn* OR belief* OR acceptance) AND (qualitative OR “focus group” OR “focus groups” OR interview*)) NOT (campaign* OR strateg* OR program* OR messag* OR intervention* OR technology OR teach* OR curriculum OR workshop* OR initiative* OR educat* OR communicat* OR information OR media OR brochure OR pamphlet OR learn* OR instruction* OR train* OR label* OR internet))*  in **Article title**  **OR**  in **Abstract**  **OR**  in **Descriptors** |
| **Search string 2:** | *(("food safety" OR "food-borne" OR foodborne OR foodbourne OR “food-bourne” OR "food handling" OR "food preparation" OR "food poisoning" OR “food hygiene” OR “safe food”) AND (consumer* OR client* OR population OR public OR people OR person OR persons OR individual OR individuals OR student OR students OR mother* OR father* OR adult OR adults OR women OR female* OR communit* OR families OR family OR household* OR domestic OR volunteer OR volunteers OR home OR homes OR campus OR university OR universities OR college*) AND (awareness OR knowledge OR practice* OR behaviour* OR behavior* OR attitude* OR perception* OR preference* OR learn* OR belief* OR acceptance) AND (qualitative OR “focus group” OR “focus groups” OR interview*))*  in **Article title**  **OR**  in **Abstract**  **OR**  in **Descriptors** |
| **Hits** | 591 |
| **Limits** | **Search string 1=**Date limit up to 2014  **Search string 2=**Date limit from 2014 to present |

| **Date** | January 8, 2016 |
| --- | --- |
| **Platform/Interface** | ProQuest |
| **Databases** | ProQuest Public Health (1972-2016) |
| **Institution** | Public Health Agency of Canada |
| **Search string 1:** | *((("food safety" OR "food-borne" OR foodborne OR foodbourne OR “food-bourne” OR "food handling" OR "food preparation" OR "food poisoning" OR “food hygiene” OR “safe food”) AND (consumer* OR client* OR population OR public OR people OR person OR persons OR individual OR individuals OR student OR students OR mother* OR father* OR adult OR adults OR women OR female* OR communit* OR families OR family OR household* OR domestic OR volunteer OR volunteers OR home OR homes OR campus OR university OR universities OR college*) AND (awareness OR knowledge OR practice* OR behaviour* OR behavior* OR attitude* OR perception* OR preference* OR learn* OR belief* OR acceptance) AND (qualitative OR “focus group” OR “focus groups” OR interview*)) NOT (campaign* OR strateg* OR program* OR messag* OR intervention* OR technology OR teach* OR curriculum OR workshop* OR initiative* OR educat* OR communicat* OR information OR media OR brochure OR pamphlet OR learn* OR instruction* OR train* OR label* OR internet))*  in **Document title**  **OR**  in **Abstract**  **OR**  in **Subject heading (all)** |
| **Search string 2:** | *(("food safety" OR "food-borne" OR foodborne OR foodbourne OR “food-bourne” OR "food handling" OR "food preparation" OR "food poisoning" OR “food hygiene” OR “safe food”) AND (consumer* OR client* OR population OR public OR people OR person OR persons OR individual OR individuals OR student OR students OR mother* OR father* OR adult OR adults OR women OR female* OR communit* OR families OR family OR household* OR domestic OR volunteer OR volunteers OR home OR homes OR campus OR university OR universities OR college*) AND (awareness OR knowledge OR practice* OR behaviour* OR behavior* OR attitude* OR perception* OR preference* OR learn* OR belief* OR acceptance) AND (qualitative OR “focus group” OR “focus groups” OR interview*))*  in **Document title**  **OR**  in **Abstract**  **OR**  in **Subject heading (all)** |
| **Hits** | 81 |
| **Limits** | **Search string 1=**Date limit up to 2014  **Search string 2=**Date limit from 2014 to present |

| **Date** | *January 6, 2016* |
| --- | --- |
| **Platform/Interface** | *EBSCOhost* |
| **Databases** | *ERIC (1966-2016)*  *CINAHL (1937-2016)* |
| **Institution** | *Ryerson University* |
| **Search string 1:** | *((("food safety" OR "food-borne" OR foodborne OR foodbourne OR “food-bourne” OR "food handling" OR "food preparation" OR "food poisoning" OR “food hygiene” OR “safe food”) AND (consumer* OR client* OR population OR public OR people OR person OR persons OR individual OR individuals OR student OR students OR mother* OR father* OR adult OR adults OR women OR female* OR communit* OR families OR family OR household* OR domestic OR volunteer OR volunteers OR home OR homes OR campus OR university OR universities OR college*) AND (awareness OR knowledge OR practice* OR behaviour* OR behavior* OR attitude* OR perception* OR preference* OR learn* OR belief* OR acceptance) AND (qualitative OR “focus group” OR “focus groups” OR interview*)) AND NOT (campaign* OR strateg* OR program* OR messag* OR intervention* OR technology OR teach* OR curriculum OR workshop* OR initiative* OR educat* OR communicat* OR information OR media OR brochure OR pamphlet OR learn* OR instruction* OR train* OR label* OR internet))*  *in* ***Title***  ***OR***  *in* ***Abstract***  *OR*  *in* ***Subject Heading*** |
| **Search string 2:** | *(("food safety" OR "food-borne" OR foodborne OR foodbourne OR “food-bourne” OR "food handling" OR "food preparation" OR "food poisoning" OR “food hygiene” OR “safe food”) AND (consumer* OR client* OR population OR public OR people OR person OR persons OR individual OR individuals OR student OR students OR mother* OR father* OR adult OR adults OR women OR female* OR communit* OR families OR family OR household* OR domestic OR volunteer OR volunteers OR home OR homes OR campus OR university OR universities OR college*) AND (awareness OR knowledge OR practice* OR behaviour* OR behavior* OR attitude* OR perception* OR preference* OR learn* OR belief* OR acceptance) AND (qualitative OR “focus group” OR “focus groups” OR interview*))*  in **Title**  **OR**  in **Abstract**  OR  in **Subject Heading** |
| **Hits** | 136 |
| **Limits** | **Search string 1=**Date limit up to 2014  **Search string 2=**Date limit from 2014 to present |
| **Date** | January 6, 2016 |
| **Platform/Interface** | ProQuest |
| **Databases** | ProQuest Dissertations & Theses A&I‎ (1743-2016) |
| **Institution** | Ryerson University |
| **Search string 1:** | *((("food safety" OR "food-borne" OR foodborne OR foodbourne OR “food-bourne” OR "food handling" OR "food preparation" OR "food poisoning" OR “food hygiene” OR “safe food”) AND (consumer* OR client* OR population OR public OR people OR person OR persons OR individual OR individuals OR student OR students OR mother* OR father* OR adult OR adults OR women OR female* OR communit* OR families OR family OR household* OR domestic OR volunteer OR volunteers OR home OR homes OR campus OR university OR universities OR college*) AND (awareness OR knowledge OR practice* OR behaviour* OR behavior* OR attitude* OR perception* OR preference* OR learn* OR belief* OR acceptance) AND (qualitative OR “focus group” OR “focus groups” OR interview*)) AND NOT (campaign* OR strateg* OR program* OR messag* OR intervention* OR technology OR teach* OR curriculum OR workshop* OR initiative* OR educat* OR communicat* OR information OR media OR brochure OR pamphlet OR learn* OR instruction* OR train* OR label* OR internet))*  in **Title**  **OR**  in **Abstract**  OR  in **Subject Heading** |
| **Search string 2:** | *(("food safety" OR "food-borne" OR foodborne OR foodbourne OR “food-bourne” OR "food handling" OR "food preparation" OR "food poisoning" OR “food hygiene” OR “safe food”) AND (consumer* OR client* OR population OR public OR people OR person OR persons OR individual OR individuals OR student OR students OR mother* OR father* OR adult OR adults OR women OR female* OR communit* OR families OR family OR household* OR domestic OR volunteer OR volunteers OR home OR homes OR campus OR university OR universities OR college*) AND (awareness OR knowledge OR practice* OR behaviour* OR behavior* OR attitude* OR perception* OR preference* OR learn* OR belief* OR acceptance) AND (qualitative OR “focus group” OR “focus groups” OR interview*))*  in **Title**  **OR**  in **Abstract**  OR  in **Subject Heading** |
| **Hits** | 35 |
| **Limits** | **Search string 1=**Date limit up to 2014  **Search string 2=**Date limit from 2014 to present |

| **Date** | January 6, 2016 |
| --- | --- |
| **Platform/Interface** | Scopus |
| **Databases** | Scopus (1823-2016) |
| **Institution** | Ryerson University |
| **Search string 1:** | *((("food safety" OR "food-borne" OR foodborne OR foodbourne OR “food-bourne” OR "food handling" OR "food preparation" OR "food poisoning" OR “food hygiene” OR “safe food”) AND (consumer* OR client* OR population OR public OR people OR person OR persons OR individual OR individuals OR student OR students OR mother* OR father* OR adult OR adults OR women OR female* OR communit* OR families OR family OR household* OR domestic OR volunteer OR volunteers OR home OR homes OR campus OR university OR universities OR college*) AND (awareness OR knowledge OR practice* OR behaviour* OR behavior* OR attitude* OR perception* OR preference* OR learn* OR belief* OR acceptance) AND (qualitative OR “focus group” OR “focus groups” OR interview*)) AND NOT (campaign* OR strateg* OR program* OR messag* OR intervention* OR technology OR teach* OR curriculum OR workshop* OR initiative* OR educat* OR communicat* OR information OR media OR brochure OR pamphlet OR learn* OR instruction* OR train* OR label* OR internet))*  in **Title**  **OR**  in **Abstract**  **OR**  **Key words** |
| **Search string 2:** | *(("food safety" OR "food-borne" OR foodborne OR foodbourne OR “food-bourne” OR "food handling" OR "food preparation" OR "food poisoning" OR “food hygiene” OR “safe food”) AND (consumer* OR client* OR population OR public OR people OR person OR persons OR individual OR individuals OR student OR students OR mother* OR father* OR adult OR adults OR women OR female* OR communit* OR families OR family OR household* OR domestic OR volunteer OR volunteers OR home OR homes OR campus OR university OR universities OR college*) AND (awareness OR knowledge OR practice* OR behaviour* OR behavior* OR attitude* OR perception* OR preference* OR learn* OR belief* OR acceptance) AND (qualitative OR “focus group” OR “focus groups” OR interview*))*  in **Title**  **OR**  in **Abstract**  **OR**  **Key words** |
| **Hits** | 381 |
| **Limits** | **Search string 1=**Date limit up to 2014  **Search string 2=**Date limit from 2014 to present |

| **Date** | January 6, 2016 |
| --- | --- |
| **Platform/Interface** | PubMed |
| **Databases** | PubMed (1950-2016) |
| **Institution** | Ryerson University |
| **Search string 1:** | *((("food safety" OR "food-borne" OR foodborne OR foodbourne OR “food-bourne” OR "food handling" OR "food preparation" OR "food poisoning" OR “food hygiene” OR “safe food”) AND (consumer* OR client* OR population OR public OR people OR person OR persons OR individual OR individuals OR student OR students OR mother* OR father* OR adult OR adults OR women OR female* OR communit* OR families OR family OR household* OR domestic OR volunteer OR volunteers OR home OR homes OR campus OR university OR universities OR college*) AND (awareness OR knowledge OR practice* OR behaviour* OR behavior* OR attitude* OR perception* OR preference* OR learn* OR belief* OR acceptance) AND (qualitative OR “focus group” OR “focus groups” OR interview*)) NOT (campaign* OR strateg* OR program* OR messag* OR intervention* OR technology OR teach* OR curriculum OR workshop* OR initiative* OR educat* OR communicat* OR information OR media OR brochure OR pamphlet OR learn* OR instruction* OR train* OR label* OR internet))*  in **All Fields**  **OR**  in **MeSH Terms** |
| **Search string 2:** | *(("food safety" OR "food-borne" OR foodborne OR foodbourne OR “food-bourne” OR "food handling" OR "food preparation" OR "food poisoning" OR “food hygiene” OR “safe food”) AND (consumer* OR client* OR population OR public OR people OR person OR persons OR individual OR individuals OR student OR students OR mother* OR father* OR adult OR adults OR women OR female* OR communit* OR families OR family OR household* OR domestic OR volunteer OR volunteers OR home OR homes OR campus OR university OR universities OR college*) AND (awareness OR knowledge OR practice* OR behaviour* OR behavior* OR attitude* OR perception* OR preference* OR learn* OR belief* OR acceptance) AND (qualitative OR “focus group” OR “focus groups” OR interview*))*  in **All Fields**  **OR**  in **MeSH Terms** |
| **Hits** | 248 |
| **Limits** | **Search string 1=**Date limit up to 2014  **Search string 2=**Date limit from 2014 to present |

Details on Grey Literature Searches and Search Verification

***List of search strings used in Google searches***

*Note: only the first 100 hits of each were screened. Google interprets each space as an “AND” Boolean operator.*

Consumer food safety qualitative

Consumer food safety focus groups

Consumer food safety interviews

***List of review articles that were hand-searched for additional potentially relevant articles***

Byrd-Bredbenner, C., Berning, J., Martin-Biggers, J., Quick, V., 2013. Food safety in home kitchens: A synthesis of the literature. Int. J. Environ. Res. Public Health 10, 4060–4085.

Evans, E.W., Redmond, E.C., 2014. Behavioral risk factors associated with listeriosis in the home: a review of consumer food safety studies. J. Food Prot. 77, 510–21.

Jacob, C., Mathiasen, L., Powell, D., 2010. Designing effective messages for microbial food safety hazards. Food Control 21, 1–6.

Nesbitt, A., Thomas, M.K., Marshall, B., Snedeker, K., Meleta, K., Watson, B., Bienefeld, M. 2014. Baseline for consumer food safety knowledge and behaviour in Canada. Food Control 38:157-73.

Redmond, E.C., Griffith, C.J., 2003. Consumer food handling in the home: a review of food safety studies. J. Food Prot. 66, 130–161.

Wilcock, A., Pun, M., Khanona, J., Aung, M. 2004. Consumer attitudes, knowledge and behaviour: A review of food safety issues. Trends Food Sci. Technol. 15:56-66.
