# Supplementary material for: Barriers and Facilitators to Safe Food Handling among Consumers: A Systematic Review and Thematic Synthesis of Qualitative Research Studies
Source: PLoS One. 2016 Dec 1;11(12):e0167695. doi: 10.1371/journal.pone.0167695 (PMC5132243; doi:10.1371/journal.pone.0167695)
Supplement: S3 File — (DOCX) [file pone.0167695.s003.docx]

S3 File – Citation List of 39 Relevant Articles

Athearn, P. N., Kendall, P. A., Hillers, V. V., Schroeder, M., Bergmann, V., Chen, G., & Medeiros, L. C. (2004). Awareness and acceptance of current food safety recommendations during pregnancy. Maternal and Child Health Journal, 8(3), 149–162.

Bearth, A., Cousin, M.-E., & Siegrist, M. (2014). Poultry consumers’ behaviour, risk perception and knowledge related to campylobacteriosis and domestic food safety. Food Control, 44, 166–176.

Bermúdez-Millán, A., Pérez-Escamilla, R., Damio, G., González, A., & Segura-Pérez, S. (2004). Food safety knowledge, attitudes, and behaviors among Puerto Rican caretakers living in Hartford, Connecticut. Journal of Food Protection, 67(3), 512–516.

Boone, K., Penner, K., Gordon, J. C., Remig, V., Harvey, L., & Clark, T. (2005). Common themes of safe-food handling behavior among mature adults. Food Protection Trends, 25(9), 706–711.

Cates, S. C., Carter-Young, H. L., Conley, S., & O’Brien, B. (2004). Pregnant women and listeriosis: preferred educational messages and delivery mechanisms. Journal of Nutrition Education and Behavior, 36(3), 121–127.

Cates, S. C., Kosa, K. M., Moore, C. M., Jaykus, L.-A., Eyck, T. A. Ten, & Cowen, P. (2007). Listeriosis Prevention for Older Adults: Effective Messages and Delivery Methods. Educational Gerontology. 33(7), 587-606.

Cates, S. C., Kosa, K. M., Teneyck, T., Jaykus, L.-A., Moore, C., & Cowen, P. (2006). Older adults’ knowledge, attitudes, and practices regarding listeriosis prevention. Food Protection Trends, 26(11), 774–785.

Chen, G., Kendall, P. A., Hillers, V. N., & Medeiros, L. C. (2010). Qualitative studies of the food safety knowledge and perceptions of transplant patients. Journal of Food Protection, 73(2), 327–335.

Coleman, H. H. (2007). Focus groups on consumer attitudes on food safety educational materials in Kentucky. Master’s thesis. University of Kentucky.

Dickinson, A., Wills, W., Meah, A., & Short, F. (2014). Food safety and older people: the Kitchen Life study. British Journal of Community Nursing, 19(5), 226, 228–232.

Dworkin, M. S., Pratap, P., Jackson, U., & Chakraborty, A. (2015). Efficacy of a culturally-tailored educational photonovella addressing prevention of meat and poultry-related food poisoning for African Americans of low socioeconomic status. Food Protection Trends, 35(3), 176–184.

Feng, Y. (2015). Aspects of food safety education and communication: consumer perception and behavior evaluation. PhD thesis. University of California, Davis.

Gettings, M. A., & Kiernan, N. E. (2001). Practices and Perceptions of Food Safety Among Seniors Who Prepare Meals at Home. Journal of Nutrition Education, 33(3), 148–154.

Godwin, S., Coppings, R., Speller-Henderson, L., & Pearson, L. (2005). Study finds consumer food safety knowledge lacking. Journal of Family and Consumer Sciences, 97(2), 40–44.

Henley, S. C., Stein, S. E., & Quinlan, J. J. (2012). Identification of unique food handling practices that could represent food safety risks for minority consumers. Journal of Food Protection, 75(11), 2050–2054.

Hoffman, E. W., Bergmann, V., Shultz, J. A., Kendall, P., Medeiros, L. C., & Hillers, V. N. (2005). Application of a five-step message development model for food safety education materials targeting people with HIV/AIDS. Journal of the American Dietetic Association, 105(10), 1597–1604.

House, E., & Coveney, J. (2013). “I mean I expect that it”s pretty safe’: Perceptions of food trust in pregnancy - implications for primary health care practice. The Australasian Medical Journal, 6(7), 358–366.

Hudson, P. K., & Hartwell, H. J. (2002). Food safety awareness of older people at home: a pilot study. The Journal of the Royal Society for the Promotion of Health, 122(3), 165–169.

Koeppl, P. T. (1998). Focus groups on barriers that limit consumers’ use of thermometers when cooking meat and poultry products. Retrieved from <http://www.fsis.usda.gov/wps/wcm/connect/864f3868-f35c-44d6-a1f4-95c3700d3cca/focusgp.pdf?MOD=AJPERES>.

Lenhart, J., Kendall, P., Medeiros, L., Doorn, J., Schroeder, M., & Sofos, J. (2008). Consumer assessment of safety and date labeling statements on ready-to-eat meat and poultry products designed to minimize risk of listeriosis. Journal of Food Protection, 71(1), 70–76.

McCarthy, M., Brennan, M., Ritson, C., & de Boer, M. (2006). Food hazard characteristics and risk reduction behaviour: the view of consumers on the island of Ireland. British Food Journal, 108(10), 875–891.

McCurdy, S. M., Hillers, V., & Cann, S. E. (2005). Consumer reaction and interest in using food thermometers when cooking small or thin meat items. Food Protection Trends, 25(11), 826–831.

Meah, A. (2013). Still blaming the consumer? Geographies of responsibility in domestic food safety practices. Critical Public Health, 24(1), 88–103.

Medeiros, L. C., Chen, G., Hillers, V. N., & Kendall, P. A. (2008). Discovery and development of educational strategies to encourage safe food handling behaviors in cancer patients. Journal of Food Protection, 71(8), 1666–1672.

Meysenburg, R., Albrecht, J. A., Litchfield, R., & Ritter-Gooder, P. K. (2014). Food safety knowledge, practices and beliefs of primary food preparers in families with young children. A mixed methods study. Appetite, 73, 121–131.

Milne, R. (2011). A focus group study of food safety practices in relation to listeriosis among the over-60s. Critical Public Health, 21(4), 485–495.

Parra, P. A., Kim, H., Shapiro, M. A., Gravani, R. B., & Bradley, S. D. (2014). Home food safety knowledge, risk perception, and practices among Mexican-Americans. Food Control, 37, 115–125.

Porticella, N., Shapiro, M. A., & Gravani, R. B. (2008). Social barriers to safer food preparation and storage practices among consumers. Paper presented at the Annual Meeting of the International Communication Association, Montreal, Quebec, Canada, May 21.

Ravarotto, L., Crovato, S., Mantovani, C., D’Este, F., Pinto, A., & Mascarello, G. (2015). Reducing microbiological risk in the kitchen: piloting consensus conference methodology as a communication strategy. Journal of Risk Research, 1–17.

Redmond, E. C. (2002). Food safety behaviour in the home: development, application and evaluation of a social marketing food safety education initiative. PhD thesis. University of Wales.

Research Triangle Institute. (2002). PR/HACCP Rule evaluation report: thermometer usage messages and delivery mechanisms for parents of young children. Retrieved from <http://www.fsis.usda.gov/wps/wcm/connect/a7cba75f-dc73-431d-b445-842e5f55fce0/rti_thermy.pdf?MOD=AJPERES>.

Roe, B., Teisl, M. F., Levy, A. S., Boyle, K., Messonnier, M. L., Riggs, T. L., … Newman, F. M. (2001). Consumers’ assessment of the food safety problem for meals prepared at home and reactions to food safety labeling. Journal of Food Products Marketing, 6(4), 9–26.

Siebert, M. M., Perry, C., O’Connell, L., Albrecht, J., Stenger, K., & Vlasin-Marty, K. (2014). A mixed methods approach to investigating food safety behavior in a sample of Native American and Hispanic caregivers of young children. Journal of Food Research, 3(5), 59–72.

Stenger, K. M., Ritter-Gooder, P. K., Perry, C., & Albrecht, J. A. (2014). A mixed methods study of food safety knowledge, practices and beliefs in Hispanic families with young children. Appetite, 83, 194–201.

Taylor, M., Kelly, M., Noël, M., Brisdon, S., Berkowitz, J., Gustafson, L., & Galanis, E. (2012). Pregnant women’s knowledge, practices, and needs related to food safety and listeriosis: a study in British Columbia. Canadian Family Physician, 58(10), 1106–1112.

Trepka, M. J., Newman, F. L., Dixon, Z., & Huffman, F. G. (2007). Food safety practices among pregnant women and mothers in the women, infants, and children program, Miami, Florida. Journal of Food Protection, 70(5), 1230–1237.

Vlasin-Marty, K. A. (2013). Food safety of Native American families with young children in Nebraska: a mixed methods study. Master’s thesis. University of Nebraska-Lincoln.

Wills, W. J., Meah, A., Dickinson, A. M., & Short, F. (2015). “I don”t think I ever had food poisoning’. A practice-based approach to understanding foodborne disease that originates in the home. Appetite, 85, 118–125.

Yarrow, L., Remig, V. M., & Higgins, M. M. (2008). Food safety awareness: concerns, practices, and openness to change of college students with health and non-health majors. Food Protection Trends, 28(8), 585–591.
