# Supplementary material for: Barriers and Facilitators to Safe Food Handling among Consumers: A Systematic Review and Thematic Synthesis of Qualitative Research Studies
Source: PLoS One. 2016 Dec 1;11(12):e0167695. doi: 10.1371/journal.pone.0167695 (PMC5132243; doi:10.1371/journal.pone.0167695)
Supplement: S1 Table — (DOCX) [file pone.0167695.s004.docx]

**S1 Table – ENTREQ Checklist**

| **No** | **Item** | **Guide and description** | **Author comments** |
| --- | --- | --- | --- |
| **1** | Aim | State the research question the synthesis addresses. | Reported in “Review approach, question, and eligibility criteria” section |
| **2** | Synthesis methodology | Identify the synthesis methodology or theoretical framework which underpins the synthesis, and describe the rationale for choice of methodology *(e.g. meta-ethnography, thematic synthesis, critical interpretive synthesis, grounded theory synthesis, realist synthesis, meta-aggregation, meta-study, framework synthesis).* | Reported in the article title and elaborated in the “analysis” section. |
| **3** | Approach to searching | Indicate whether the search was pre-planned (*comprehensive search strategies to seek all available studies)* or iterative (*to seek all available concepts until they theoretical saturation is achieved)*. | Reported in “Search strategy” section |
| **4** | Inclusion criteria | Specify the inclusion/exclusion criteria *(e.g. in terms of population, language, year limits, type of publication, study type).* | Reported in “Review approach, question, and eligibility criteria” and “Search strategy” sections |
| **5** | Data sources | Describe the information sources used (e.g. *electronic databases (MEDLINE, EMBASE, CINAHL, psycINFO, Econlit), grey literature databases (digital thesis, policy reports), relevant organisational websites, experts, information specialists, generic web searches (Google Scholar) hand searching, reference lists)* and when the searches conducted; provide the rationale for using the data sources. | Reported in “Search strategy” section, with additional details as supplementary information |
| **6** | Electronic Search strategy | Describe the literature search *(e.g. provide electronic search strategies with population terms, clinical or health topic terms, experiential or social phenomena related terms, filters for qualitative research, and search limits)*. | Reported in “Search strategy” section, with additional details as supplementary information |
| **7** | Study screening methods | Describe the process of study screening and sifting *(e.g. title, abstract and full text review, number of independent reviewers who screened studies).* | Reported in “Relevance screening, data extraction, and quality assessment” section |
| **8** | Study characteristics | Present the characteristics of the included studies *(e.g. year of publication, country, population, number of participants, data collection, methodology, analysis, research questions).* | Reported in “Study characteristics” section and Table 2, with additional details as supplementary information |
| **9** | Study selection results | Identify the number of studies screened and provide reasons for study exclusion *(e.g. for comprehensive searching, provide numbers of studies screened and reasons for exclusion indicated in a figure/flowchart; for iterative searching describe reasons for study exclusion and inclusion based on modifications to the research question and/or contribution to theory development).* | Reported in “Study characteristics” section and Figure 1 |
| **10** | Rationale for appraisal | Describe the rationale and approach used to appraise the included studies or selected findings *(e.g. assessment of conduct (validity and robustness), assessment of reporting (transparency), assessment of content and utility of the findings).* | Reported in “Relevance screening, data extraction, and quality assessment” section |
| **11** | Appraisal items | State the tools, frameworks and criteria used to appraise the studies or selected findings *(e.g. Existing tools: CASP, QARI, COREQ, Mays and Pope* [[25](http://bmcmedresmethodol.biomedcentral.com/articles/10.1186/1471-2288-12-181#CR25)]*; reviewer developed tools; describe the domains assessed: research team, study design, data analysis and interpretations, reporting).* | Reported in “Relevance screening, data extraction, and quality assessment” section, with full tool available as supplementary information |
| **12** | Appraisal process | Indicate whether the appraisal was conducted independently by more than one reviewer and if consensus was required. | Reported in “Review management” section |
| **13** | Appraisal results | Present results of the quality assessment and indicate which articles, if any, were weighted/excluded based on the assessment and give the rationale. | Reported in “Study characteristics” section and Table 1, with additional details available as supplementary information |
| **14** | Data extraction | Indicate which sections of the primary studies were analysed and how were the data extracted from the primary studies? *(e.g. all text under the headings “results /conclusions” were extracted electronically and entered into a computer software).* | Reported in “Analysis” section |
| **15** | Software | State the computer software used, if any. | Reported in “Analysis” section |
| **16** | Number of reviewers | Identify who was involved in coding and analysis. | Reported in “Analysis” section |
| **17** | Coding | Describe the process for coding of data *(e.g. line by line coding to search for concepts).* | Reported in “Analysis” section |
| **18** | Study comparison | Describe how were comparisons made within and across studies *(e.g. subsequent studies were coded into pre-existing concepts, and new concepts were created when deemed necessary).* | Reported in “Analysis” section |
| **19** | Derivation of themes | Explain whether the process of deriving the themes or constructs was inductive or deductive. | Reported in “Analysis” section |
| **20** | Quotations | Provide quotations from the primary studies to illustrate themes/constructs, and identify whether the quotations were participant quotations of the author’s interpretation. | Reported in “Descriptive themes” section |
| **21** | Synthesis output | Present rich, compelling and useful results that go beyond a summary of the primary studies (e.g. *new interpretation, models of evidence, conceptual models, analytical framework, development of a new theory or construct).* | Reported in “Analytical themes” section |
