# Supplementary material for: Barriers and Facilitators to Safe Food Handling among Consumers: A Systematic Review and Thematic Synthesis of Qualitative Research Studies
Source: PLoS One. 2016 Dec 1;11(12):e0167695. doi: 10.1371/journal.pone.0167695 (PMC5132243; doi:10.1371/journal.pone.0167695)
Supplement: S2 Table — (DOCX) [file pone.0167695.s005.docx]

S2 Table – Detailed CERQual Assessment Table

| **Finding** | **Adequacy of data** | **Relevance** | **Coherence** | **Methodological limitations** | **Overall confidence** | **References^1^** |
| --- | --- | --- | --- | --- | --- | --- |
| **Confidence and perceived risk** |  |  |  |  |  |  |
| **Barrier**: Lack of self-perceived risk due to confidence in own practices | **No concerns:** rich data from 28 studies (30 articles) | **No concerns** | **No concerns** | **Minor concerns:** methodological limitations in some of the represented studies | **High:** finding is supported by 28 studies with rich data, and minor methodological concerns | (Athearn et al., 2004; Bearth et al., 2014; Boone et al., 2005; Cates et al., 2007, 2006, 2004; Chen et al., 2010; Dickinson et al., 2014; Feng, 2015; Gettings and Kiernan, 2001; Henley et al., 2012; Hoffman et al., 2005; House and Coveney, 2013; Hudson and Hartwell, 2002; Koeppl, 1998; McCarthy et al., 2006; Meah, 2013; Meysenburg et al., 2014; Parra et al., 2014; Porticella et al., 2008; Ravarotto et al., 2015; Redmond, 2002; Research Triangle Institute, 2002; Roe et al., 2001; Siebert et al., 2014; Stenger et al., 2014; Trepka et al., 2007; Vlasin-Marty, 2013; Wills et al., 2015; Yarrow et al., 2008) |
| **Barrier:** Belief in higher risk due to food prepared and handled by others | **No concerns:** rich data from 21 studies (22 articles) | **No concerns** | **Minor concerns:** some inconsistencies in whether consumers trust some stakeholders more than others (e.g. local butcher) | **Minor concerns:** methodological limitations in some of the represented studies | **High:** finding is supported by 21 studies with rich data, and minor methodological and coherence concerns | (Bearth et al., 2014; Boone et al., 2005; Cates et al., 2006; Chen et al., 2010; Coleman, 2007; Dickinson et al., 2014; Feng, 2015; Godwin et al., 2005; Hoffman et al., 2005; Koeppl, 1998; Lenhart et al., 2008; McCarthy et al., 2006; Meah, 2013; Medeiros et al., 2008; Redmond, 2002; Roe et al., 2001; Siebert et al., 2014; Stenger et al., 2014; Trepka et al., 2007; Vlasin-Marty, 2013; Wills et al., 2015; Yarrow et al., 2008) |
| **Barrier:** Not concerned about food safety because they have never previously experienced illness from food prepared at home | **Moderate concerns:** supported by 17 studies, with limited depth of data provided in most studies | **No concerns** | **No concerns** | **Moderate concerns:** methodological limitations for multiple criteria across several represented studies | **Moderate:** finding is supported by 17 studies with limited data richness and moderate methodological concerns | (Athearn et al., 2004; Bearth et al., 2014; Cates et al., 2006; Coleman, 2007; Dickinson et al., 2014; Feng, 2015; Gettings and Kiernan, 2001; Hudson and Hartwell, 2002; Koeppl, 1998; Meah, 2013; Meysenburg et al., 2014; Porticella et al., 2008; Research Triangle Institute, 2002; Roe et al., 2001; Siebert et al., 2014; Vlasin-Marty, 2013; Yarrow et al., 2008) |
| **Barrier:** Confidence in the food system to provide safe food | **Minor concerns:** supported by 17 studies (18 articles), some with limited depth of data provided | **No concerns** | **Minor concerns:** some inconsistencies in extent of trust in food system reported among consumers | **Minor concerns:** methodological limitations in some of the represented studies | **Moderate:** finding is supported by 17 studies with minor adequacy of data, coherence and methodological concerns | (Athearn et al., 2004; Bearth et al., 2014; Cates et al., 2006; Chen et al., 2010; Dickinson et al., 2014; Feng, 2015; Hoffman et al., 2005; House and Coveney, 2013; Koeppl, 1998; Lenhart et al., 2008; Meah, 2013; Ravarotto et al., 2015; Redmond, 2002; Roe et al., 2001; Taylor et al., 2012; Vlasin-Marty, 2013; Wills et al., 2015; Yarrow et al., 2008) |
| **Barrier:** Belief that foodborne illness is outside of consumers’ control | **Substantial concerns:** supported by 5 studies with limited depth of data provided | **No concerns** | **No concerns** | **Minor concerns:** methodological limitations in some of the represented studies | **Low:** finding is supported by only 5 studies with limited data richness | (Koeppl, 1998; Meysenburg et al., 2014; Redmond, 2002; Roe et al., 2001; Vlasin-Marty, 2013) |
| **Facilitator:** Concern for dependents (e.g. children, elderly family members) at higher risk of foodborne illness and for whom they prepare food | **No concerns**: rich data from 18 studies (19 articles) | **No concerns** | **No concerns** | **Minor concerns:** methodological limitations in some of the represented studies | **High:** finding is supported by 18 studies with rich data, and minor methodological concerns | (Athearn et al., 2004; Cates et al., 2007, 2006, 2004; Coleman, 2007; Feng, 2015; Godwin et al., 2005; House and Coveney, 2013; Koeppl, 1998; McCarthy et al., 2006; McCurdy et al., 2005; Meysenburg et al., 2014; Porticella et al., 2008; Redmond, 2002; Research Triangle Institute, 2002; Siebert et al., 2014; Trepka et al., 2007; Vlasin-Marty, 2013; Wills et al., 2015) |
| **Facilitator:** Belonging to certain high-risk groups (e.g. immuno-compromised, first-time pregnant women) increases willingness to change food handling behaviours | **Minor concerns:** supported by 14 studies (15 articles), some with limited depth of data provided | **No concerns** | **Moderate concern:** some inconsistencies reported in desire to change some practices | **Minor concerns:** methodological limitations in some of the represented studies | **Moderate:** finding is supported by 14 studies, with minor adequacy of data and methodological concerns, and moderate coherence concerns | (Athearn et al., 2004; Boone et al., 2005; Cates et al., 2007, 2004; Chen et al., 2010; Coleman, 2007; Dickinson et al., 2014; Feng, 2015; House and Coveney, 2013; Medeiros et al., 2008; Milne, 2011; Siebert et al., 2014; Taylor et al., 2012; Trepka et al., 2007; Wills et al., 2015) |
| **Facilitator:** Concern about the cost and inconvenience of foodborne illness | **No concerns:** rich data from 29 studies (31 articles) | **No concerns** | **Moderate concerns:** some inconsistencies reported in perceived level of concern | **Minor concerns:** methodological limitations in some of the represented studies | **Moderate:** finding is supported by 29 studies with rich data and minor methodological concerns, but some inconsistencies reported | (Athearn et al., 2004; Boone et al., 2005; Cates et al., 2007, 2006, 2004; Chen et al., 2010; Coleman, 2007; Dickinson et al., 2014; Feng, 2015; Gettings and Kiernan, 2001; Godwin et al., 2005; Hoffman et al., 2005; House and Coveney, 2013; Hudson and Hartwell, 2002; Koeppl, 1998; McCarthy et al., 2006; McCurdy et al., 2005; Meah, 2013; Medeiros et al., 2008; Meysenburg et al., 2014; Milne, 2011; Porticella et al., 2008; Redmond, 2002; Research Triangle Institute, 2002; Roe et al., 2001; Siebert et al., 2014; Stenger et al., 2014; Trepka et al., 2007; Vlasin-Marty, 2013; Wills et al., 2015; Yarrow et al., 2008) |
| **Facilitator:** Higher concern among those who have previously experienced foodborne illness or know someone who has, and believe it was due to food prepared at home | **Moderate concerns:** supported by 11 studies (12 articles) with limited depth of data provided in most studies | **No concerns** | **Moderate concerns:** some inconsistencies reported in potential impact on behaviour change | **Minor concerns:** methodological limitations in some of the represented studies | **Low:** finding is supported by 11 studies with minor methodological concerns, and moderate data richness and coherence concerns | (Athearn et al., 2004; Cates et al., 2007, 2006; Coleman, 2007; Godwin et al., 2005; Koeppl, 1998; McCurdy et al., 2005; Meysenburg et al., 2014; Porticella et al., 2008; Redmond, 2002; Research Triangle Institute, 2002; Vlasin-Marty, 2013) |
| **Knowledge-behaviour gap** |  |  |  |  |  |  |
| **Barrier:** Lack of knowledge and misconceptions about some recommended safe food handling practices | **No concerns:** rich data from 34 studies (36 articles) | **No concerns** | **No concerns** | **Minor concerns:** methodological limitations in some of the represented studies | **High:** finding is supported by 34 studies with rich data, and minor methodological concerns | (Athearn et al., 2004; Bearth et al., 2014; Bermúdez-Millán et al., 2004; Boone et al., 2005; Cates et al., 2007, 2006, 2004; Chen et al., 2010; Coleman, 2007; Dickinson et al., 2014; Dworkin et al., 2015; Feng, 2015; Gettings and Kiernan, 2001; Godwin et al., 2005; Hoffman et al., 2005; Hudson and Hartwell, 2002; Koeppl, 1998; Lenhart et al., 2008; McCarthy et al., 2006; McCurdy et al., 2005; Meah, 2013; Medeiros et al., 2008; Meysenburg et al., 2014; Parra et al., 2014; Porticella et al., 2008; Ravarotto et al., 2015; Redmond, 2002; Research Triangle Institute, 2002; Roe et al., 2001; Siebert et al., 2014; Stenger et al., 2014; Taylor et al., 2012; Trepka et al., 2007; Vlasin-Marty, 2013; Wills et al., 2015; Yarrow et al., 2008) |
| **Barrier:** Disagreement with some recommendations for safe food handling due to conflicting beliefs and perceptions | **No concerns:** rich data from 22 studies (24 articles) | **No concerns** | **Minor concerns:** inconsistencies and mixed consumer perceptions reported for some practices | **Minor concerns:** methodological limitations in some of the represented studies | **High:** finding is supported by 22 studies, with minor coherence and methodological concerns | (Athearn et al., 2004; Boone et al., 2005; Cates et al., 2007, 2006; Chen et al., 2010; Coleman, 2007; Dickinson et al., 2014; Feng, 2015; Gettings and Kiernan, 2001; Hoffman et al., 2005; House and Coveney, 2013; Koeppl, 1998; Lenhart et al., 2008; McCurdy et al., 2005; Meah, 2013; Medeiros et al., 2008; Parra et al., 2014; Porticella et al., 2008; Redmond, 2002; Research Triangle Institute, 2002; Roe et al., 2001; Trepka et al., 2007; Wills et al., 2015; Yarrow et al., 2008) |
| **Barrier:** Some unsafe food handling behaviours followed despite being aware of recommended practices | **Moderate concerns:** supported by 13 studies (14 articles) , with limited depth of data provided in most studies | **No concerns** | **No concerns** | **Minor concerns:** methodological limitations in some of the represented studies | **Moderate:** finding is supported by 13 studies, with moderate data richness and minor methodological concerns | (Athearn et al., 2004; Bearth et al., 2014; Cates et al., 2006, 2004; Coleman, 2007; Dickinson et al., 2014; Feng, 2015; House and Coveney, 2013; Hudson and Hartwell, 2002; Meah, 2013; Porticella et al., 2008; Vlasin-Marty, 2013; Wills et al., 2015; Yarrow et al., 2008) |
| **Facilitator:** Some recommended practices followed, often from the perspective of “common sense” and general hygiene than for food safety reasons | **No concerns:** rich data from 32 studies (34 articles) | **No concerns** | **No concerns** | **Minor concerns:** methodological limitations in some of the represented studies | **High:** finding is supported by 32 studies with rich data, and minor methodological concerns | (Athearn et al., 2004; Bermúdez-Millán et al., 2004; Boone et al., 2005; Cates et al., 2007, 2006, 2004; Chen et al., 2010; Coleman, 2007; Dickinson et al., 2014; Dworkin et al., 2015; Feng, 2015; Gettings and Kiernan, 2001; Godwin et al., 2005; Henley et al., 2012; House and Coveney, 2013; Hudson and Hartwell, 2002; Koeppl, 1998; Lenhart et al., 2008; McCarthy et al., 2006; Meah, 2013; Medeiros et al., 2008; Milne, 2011; Parra et al., 2014; Porticella et al., 2008; Ravarotto et al., 2015; Redmond, 2002; Research Triangle Institute, 2002; Roe et al., 2001; Siebert et al., 2014; Stenger et al., 2014; Trepka et al., 2007; Vlasin-Marty, 2013; Wills et al., 2015; Yarrow et al., 2008) |
| **Facilitator:** Willingness to learn more about food safety | **Moderate concerns:** supported by 15 studies (16 articles), with limited depth of data provided in most studies | **Moderate concerns:** most supporting studies were in high-risk consumer groups, wider applicability unclear | **Moderate concerns:** some inconsistencies reported in desire to learn more, how information was requested, and the potential impact | **Minor concerns:** methodological limitations in some of the represented studies | **Low:** finding is supported by 15 studies with limited data richness and moderate concerns for relevance and coherence | (Athearn et al., 2004; Bermúdez-Millán et al., 2004; Boone et al., 2005; Cates et al., 2007, 2006; Chen et al., 2010; Feng, 2015; Godwin et al., 2005; Hoffman et al., 2005; McCurdy et al., 2005; Medeiros et al., 2008; Porticella et al., 2008; Stenger et al., 2014; Taylor et al., 2012; Vlasin-Marty, 2013; Yarrow et al., 2008) |
| **Habits and heuristics** |  |  |  |  |  |  |
| **Barrier:** Food handling behaviours are routine and unconscious, influenced by past experiences, and difficult to change | **No concerns:** rich data from 29 studies (30 articles) | **No concerns** | **No concerns** | **Minor concerns:** methodological limitations in some of the represented studies | **High:** finding is supported by 29 studies with rich data, and minor methodological concerns | (Athearn et al., 2004; Bearth et al., 2014; Bermúdez-Millán et al., 2004; Boone et al., 2005; Cates et al., 2006; Coleman, 2007; Dickinson et al., 2014; Dworkin et al., 2015; Feng, 2015; Gettings and Kiernan, 2001; Godwin et al., 2005; Henley et al., 2012; Hoffman et al., 2005; House and Coveney, 2013; Koeppl, 1998; McCurdy et al., 2005; Meah, 2013; Medeiros et al., 2008; Meysenburg et al., 2014; Milne, 2011; Parra et al., 2014; Porticella et al., 2008; Ravarotto et al., 2015; Redmond, 2002; Research Triangle Institute, 2002; Roe et al., 2001; Siebert et al., 2014; Trepka et al., 2007; Wills et al., 2015) |
| **Barrier:** Various heuristics and “rules of thumb” used (e.g. sensory checks) when handling and preparing food | **No concerns:** rich data from 31 studies (33 articles) | **No concerns** | **No concerns** | **Minor concerns:** methodological limitations in some of the represented studies | **High:** finding is supported by 31 studies with rich data, and minor methodological concerns | (Athearn et al., 2004; Bearth et al., 2014; Bermúdez-Millán et al., 2004; Boone et al., 2005; Cates et al., 2007, 2006; Coleman, 2007; Dickinson et al., 2014; Dworkin et al., 2015; Feng, 2015; Gettings and Kiernan, 2001; Godwin et al., 2005; Henley et al., 2012; Hoffman et al., 2005; House and Coveney, 2013; Hudson and Hartwell, 2002; Koeppl, 1998; Lenhart et al., 2008; McCarthy et al., 2006; McCurdy et al., 2005; Meah, 2013; Medeiros et al., 2008; Meysenburg et al., 2014; Parra et al., 2014; Porticella et al., 2008; Research Triangle Institute, 2002; Roe et al., 2001; Siebert et al., 2014; Stenger et al., 2014; Trepka et al., 2007; Vlasin-Marty, 2013; Wills et al., 2015) |
| **Practical and lifestyle constraints** |  |  |  |  |  |  |
| **Barrier:** Inconvenience, lack of time, laziness and negligence contribute to unsafe practices | **No concerns:** rich data from 25 studies (26 articles) | **No concerns** | **No concerns** | **Minor concerns:** methodological limitations in some of the represented studies | **High:** finding is supported by 25 studies with rich data, and minor methodological concerns | (Athearn et al., 2004; Bearth et al., 2014; Boone et al., 2005; Cates et al., 2007, 2006; Chen et al., 2010; Coleman, 2007; Feng, 2015; Gettings and Kiernan, 2001; Henley et al., 2012; Hoffman et al., 2005; Hudson and Hartwell, 2002; Koeppl, 1998; McCarthy et al., 2006; McCurdy et al., 2005; Medeiros et al., 2008; Meysenburg et al., 2014; Porticella et al., 2008; Ravarotto et al., 2015; Redmond, 2002; Research Triangle Institute, 2002; Siebert et al., 2014; Stenger et al., 2014; Trepka et al., 2007; Vlasin-Marty, 2013; Yarrow et al., 2008) |
| **Barrier:** Distractions in the kitchen interfere with safe food handling | **Moderate concerns:** supported by 10 studies, with limited depth provided in most studies | **No concerns** | **No concerns** | **Minor concerns:** methodological limitations in some of the represented studies | **Moderate:** finding is supported by 10 studies with limited data richness, and minor methodological concerns | (Bearth et al., 2014; Coleman, 2007; Feng, 2015; McCarthy et al., 2006; Meysenburg et al., 2014; Parra et al., 2014; Siebert et al., 2014; Stenger et al., 2014; Vlasin-Marty, 2013; Wills et al., 2015) |
| **Barrier:** Lack of proper resources and tools to facilitate safe food handling | **Minor concerns:** supported by 19 studies (20 articles), with limited depth provided in some studies | **Minor concerns:** focused on a lack of thermometer ownership | **No concerns** | **Minor concerns:** methodological limitations in some of the represented studies | **Moderate:** finding is supported by 19 studies, with minor data richness, relevance, and methodological concerns | (Athearn et al., 2004; Bearth et al., 2014; Boone et al., 2005; Cates et al., 2007, 2006; Chen et al., 2010; Dickinson et al., 2014; Feng, 2015; Gettings and Kiernan, 2001; Henley et al., 2012; Koeppl, 1998; Medeiros et al., 2008; Milne, 2011; Parra et al., 2014; Porticella et al., 2008; Siebert et al., 2014; Stenger et al., 2014; Trepka et al., 2007; Vlasin-Marty, 2013; Yarrow et al., 2008) |
| **Barrier:** Inability to access or use resources due to kitchen layout or physical constraints | **Substantial concerns:** supported by 5 studies (6 articles) with limited depth of data provided | **No concerns** | **No concerns** | **Moderate concerns:** methodological limitations for multiple criteria across several represented studies | **Low:** finding is supported by only 5 studies with limited data richness and moderate methodological concerns | (Dickinson et al., 2014; Gettings and Kiernan, 2001; Hudson and Hartwell, 2002; Koeppl, 1998; McCurdy et al., 2005; Wills et al., 2015) |
| **Barrier:** Safe food handling is another “burden” for some high-risk groups of consumers | **Substantial concerns:** supported by 5 studies with limited depth of data provided | **Moderate concerns:** studies reported only for certain groups of consumers in specific settings, wider applicability unclear | **No concerns** | **Moderate concerns:** methodological limitations for multiple criteria across several represented studies | **Low:** finding is supported by only 5 studies, with limited data richness, limited applicability to other populations, and moderate methodological concerns | (Athearn et al., 2004; Feng, 2015; Hoffman et al., 2005; Medeiros et al., 2008; Milne, 2011) |
| **Barrier:** Reluctance to dispose of expired food among older adults | **Substantial concerns:** supported by 5 studies (6 articles) with limited depth of data provided | **Moderate concerns:** finding almost exclusively reported among older adults, wider applicability unclear | **Moderate concerns:** finding was not consistently identified in studies of older adults | **Minor concerns:** methodological limitations in some of the represented studies | **Low:** finding is supported by only 5 studies, with limited data richness, limited applicability to other populations, and moderate coherence concerns | (Cates et al., 2007; Dickinson et al., 2014; Gettings and Kiernan, 2001; Meah, 2013; Milne, 2011; Wills et al., 2015) |
| **Barrier:** Unique challenges for older adults and low-income households | **Substantial concerns:** supported by 3 studies with limited data richness | **Moderate concerns:** studies reported only for certain groups of consumers in specific settings, wider applicability unclear | **Moderate concerns:** finding was not consistently identified across studies of older adults and low-income consumers | **Moderate concerns:** methodological limitations for multiple criteria across several represented studies | **Low:** finding is supported by only 3 studies, with limited data richness, limited applicability to other populations, and moderate coherence and methodological concerns | (Henley et al., 2012; Hudson and Hartwell, 2002; Milne, 2011) |
| **Facilitator:** Willingness to change behaviours if practical constraints were minimized or removed | **Minor concerns:** supported by 12 studies, with limited depth provided in some studies | **Minor concerns:** most supporting research focused on provision of a thermometer | **No concerns** | **Moderate concerns:** methodological limitations for multiple criteria across several represented studies | **Moderate:** finding is supported by 12 studies with minor data richness and relevance concerns, and moderate methodological concerns | (Athearn et al., 2004; Bermúdez-Millán et al., 2004; Boone et al., 2005; Cates et al., 2006; Feng, 2015; Henley et al., 2012; Koeppl, 1998; McCurdy et al., 2005; Milne, 2011; Porticella et al., 2008; Research Triangle Institute, 2002; Yarrow et al., 2008) |
| **Food preferences** |  |  |  |  |  |  |
| **Barrier:** Food choices driven by quality, perceived health benefits, and convenience over considerations for food safety | **Minor concerns:** supported by 17 studies (19 articles), with limited depth provided in some studies | **No concerns** | **No concerns** | **Minor concerns:** methodological limitations in some of the represented studies | **High:** finding is supported by 17 studies, with minor data richness and methodological concerns | (Athearn et al., 2004; Bearth et al., 2014; Boone et al., 2005; Cates et al., 2007, 2006; Chen et al., 2010; Dickinson et al., 2014; Feng, 2015; House and Coveney, 2013; McCarthy et al., 2006; Medeiros et al., 2008; Meysenburg et al., 2014; Parra et al., 2014; Takeuchi et al., 2006; Trepka et al., 2007; Wills et al., 2015; Yarrow et al., 2008) |
| **Facilitator:** Preferred quality characteristics of safely prepared foods | **Moderate concerns:** supported by 10 studies, with limited depth provided in most studies | **Minor concerns:** most supporting research focused only on thermometer use when cooking meat | **No concerns** | **Moderate concerns:** methodological limitations for multiple criteria across several represented studies | **Low:** finding is supported by 10 studies with limited data richness and moderate methodological concerns | (Athearn et al., 2004; Boone et al., 2005; Cates et al., 2006, 2004; Chen et al., 2010; Henley et al., 2012; Koeppl, 1998; McCurdy et al., 2005; Research Triangle Institute, 2002; Roe et al., 2001) |
| **Societal and social influences** |  |  |  |  |  |  |
| **Barrier:** Negative social acceptability of some recommended practices | **Substantial concerns:** supported by 7 studies, with limited depth and quotes provided | **Minor concerns:** most supporting research focused only on thermometer use when cooking meat | **Moderate concerns:** some inconsistencies reported in terms of whether social acceptability would positively or negatively affect behaviours | **Moderate concerns:** methodological limitations for multiple criteria across several represented studies | **Low:** finding is supported by 7 studies, with limited richness in data, and some relevance, coherence, and methodological concerns | (Bearth et al., 2014; Feng, 2015; Koeppl, 1998; McCurdy et al., 2005; Porticella et al., 2008; Research Triangle Institute, 2002; Trepka et al., 2007) |
| **Barrier:** Cultural traditions associated with some unsafe food handling practices | **Minor concerns:** supported by 9 studies in diversity of populations, but limited depth provided in some studies | **No concerns** | **No concerns** | **Minor concerns:** methodological limitations in some of the represented studies | **Moderate:** finding is supported by 9 studies with limited data richness and minor methodological concerns, but with diversity of populations represented | (Coleman, 2007; Godwin et al., 2005; Henley et al., 2012; Parra et al., 2014; Siebert et al., 2014; Stenger et al., 2014; Vlasin-Marty, 2013) |
| **Barrier:** Unsafe practices learned through family, friends, and social networks | **Minor concerns:** supported by 13 studies, with limited depth provided in some studies | **No concerns** | **Minor concerns:** some studies report that social networks could also propagate safe food handling practices | **Moderate concerns:** methodological limitations for multiple criteria across several represented studies | **Moderate:** finding is supported by 13 studies with minor data richness concerns and moderate methodological concerns | (Athearn et al., 2004; Coleman, 2007; Feng, 2015; Gettings and Kiernan, 2001; Godwin et al., 2005; Koeppl, 1998; McCarthy et al., 2006; Meah, 2013; Ravarotto et al., 2015; Trepka et al., 2007; Vlasin-Marty, 2013; Wills et al., 2015; Yarrow et al., 2008) |
| **Facilitator:** Healthcare providers and extension services as trusted sources of food safety information | **No concerns**: rich data from 18 studies (19 references) | **No concerns** | **Minor concerns:** some studies reported a failure of healthcare professionals to provide sufficient information in some cases (e.g. pregnant women) | **Minor concerns:** methodological limitations in some of the represented studies | **High:** finding is supported by 18 studies with rich data, and minor methodological and coherence concerns | (Athearn et al., 2004; Bermúdez-Millán et al., 2004; Cates et al., 2007, 2006, 2004; Chen et al., 2010; Coleman, 2007; Gettings and Kiernan, 2001; House and Coveney, 2013; Koeppl, 1998; McCurdy et al., 2005; Medeiros et al., 2008; Porticella et al., 2008; Research Triangle Institute, 2002; Siebert et al., 2014; Stenger et al., 2014; Taylor et al., 2012; Trepka et al., 2007; Vlasin-Marty, 2013) |
| **Facilitator:** Media stories and coverage increase food safety awareness | **No concerns**: rich data from 16 studies | **No concerns** | **Moderate concerns:** some inconsistencies reported in perceived role and impact of media (e.g. concern for media “hype” and credibility) | **Minor concerns:** methodological limitations in some of the represented studies | **Moderate:** finding is supported by 16 studies with rich data and minor methodological concerns, but with moderate coherence concerns | (Athearn et al., 2004; Boone et al., 2005; Cates et al., 2004; Chen et al., 2010; Coleman, 2007; Gettings and Kiernan, 2001; Godwin et al., 2005; Koeppl, 1998; Lenhart et al., 2008; Meah, 2013; Parra et al., 2014; Redmond, 2002; Research Triangle Institute, 2002; Roe et al., 2001; Siebert et al., 2014; Trepka et al., 2007) |

^1^ For full citation information for each reference, refer to the citation list in S3 File.
